# Competition shapes the landscape of X-chromosome-linked genetic diversity

**DOI:** 10.1038/s41588-024-01840-5

**Published:** 2024-07-26

**Authors:** Teresa Buenaventura, Hakan Bagci, Ilinca Patrascan, Joshua J. Graham, Kelsey D. Hipwell, Roel Oldenkamp, James W. D. King, Jesus Urtasun, George Young, Daniel Mouzo, David Gomez-Cabrero, Benjamin D. Rowland, Daniel Panne, Amanda G. Fisher, Matthias Merkenschlager

**Affiliations:** 1 MRC LMS, Institute of Clinical Sciences, Faculty of Medicine, Imperial College London, London, UK; 2 Leicester Institute of Structural and Chemical Biology, Department of Molecular and Cell Biology, University of Leicester, Leicester, UK; 3 Division of Cell Biology, Netherlands Cancer Institute, Amsterdam, The Netherlands; 4 Translational Bioinformatics Unit, Navarrabiomed, Universidad Pública de Navarra (UPNA), Instituto de Investigación Sanitaria de Navarra (IdiSNA), Pamplona, Spain; 5 Bioscience Program, Biological and Environmental Sciences and Engineering Division (BESE), King Abdullah University of Science and Technology KAUST, Thuwal, Saudi Arabia; 6 Department of Biochemistry, University of Oxford, Oxford, UK

**Keywords:** Epigenetics, Cell biology, Stem cells

## Abstract

X chromosome inactivation (XCI) generates clonal heterogeneity within XX individuals. Combined with sequence variation between human X chromosomes, XCI gives rise to intra-individual clonal diversity, whereby two sets of clones express mutually exclusive sequence variants present on one or the other X chromosome. Here we ask whether such clones merely co-exist or potentially interact with each other to modulate the contribution of X-linked diversity to organismal development. Focusing on X-linked coding variation in the human *STAG2* gene, we show that *Stag2*^variant^ clones contribute to most tissues at the expected frequencies but fail to form lymphocytes in *Stag2*^WT^ *Stag2*^variant^ mouse models. Unexpectedly, the absence of *Stag2*^variant^ clones from the lymphoid compartment is due not solely to cell-intrinsic defects but requires continuous competition by *Stag2*^WT^ clones. These findings show that interactions between epigenetically diverse clones can operate in an XX individual to shape the contribution of X-linked genetic diversity in a cell-type-specific manner.

## Abstract (web summary)

Clones expressing variants of the X-chromosome-linked *STAG2* gene only form lymphocytes in the absence of *Stag2*^WT^ clones. Interactions between epigenetic clones can thus modulate the impact of X-chromosome-linked genetic diversity in a cell-type-specific manner.

## Main

**Fig. 1:**
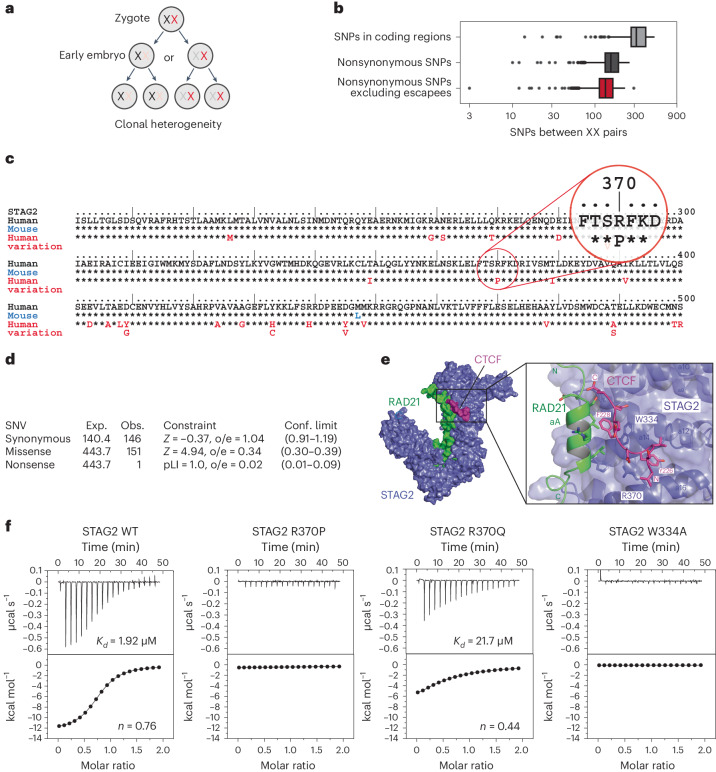
Sequence variation in the human X-linked *STAG2* gene disrupts cohesin–CTCF binding. **a**, XCI and its epigenetic propagation give rise to intra-individual clonal heterogeneity **b**, The number of SNPs between any two X chromosomes across 2,504 individuals from phase 3 of the 1000 Genomes Project. Box plots show the median, upper and lower quartiles, and whiskers show the extremes. **c**, Partial STAG2 protein sequence alignment of human (black) and mouse (blue), as well as sequence variation in the human population (red, gnomAD v2.1.1). gnomAD variant X-123185062—G-C (GRCh37) is highlighted. Extended Data Fig. 2 shows an alignment of the full STAG2 protein sequence and additional details. **d**, Constraint matrix based on canonical ENSEMBL transcript ENST000003218089.9. *Z* = 4.94, o/e = 0.34; 0.30–0.39 (gnomAD v2.1.1). **e**, Structure of the interface between cohesin (STAG2/RAD21) and CTCF^16^. **f**, Impact of STAG2 variants on cohesin (STAG2/RAD21) interactions with CTCF as determined by isothermal calorimetry (see Extended Data Fig. 3a,b for the characterization of proteins used in isothermal calorimetry experiments). SNV, single-nucleotide variant. Source data

Eutherian mammals such as humans and mice compensate for differences in X-linked gene dosage between males and females by X chromosome inactivation^1^ (XCI; Fig. 1a). In XX embryos, each cell randomly chooses one of its two X chromosomes for inactivation, which results in the silencing of the majority of genes on that chromosome^1–4^. XX embryos therefore resemble mixtures of clones expressing genes from either their maternal or paternal X chromosome. The identities of the active (Xa) and inactive (Xi) X chromosomes are clonally propagated through organismal development by epigenetic mechanisms^5,6^. Hence, XX individuals are clonally heterogeneous as a result of XCI and its propagation.

Human population shows extensive genetic diversity, including single-nucleotide polymorphisms^7^ (SNPs), which occur at comparable frequencies on autosomes and X chromosomes^8^ (Supplementary Table 1). The human X chromosome harbors >600 protein-coding genes annotated in OMIM, the Online Catalog of Human Genes and Genetic Disorders^9^. Together, these genes contain ~400k nonsynonymous SNPs that change their coding potential^10^, indicating extensive variation between human X chromosomes. This variation, combined with XCI and its epigenetic propagation, gives rise to intra-individual clonal diversity in XX individuals.

Given that X-linked intra-individual diversity is widespread among XX individuals, it is of interest to consider its potential significance for organismal development. What is known so far is that stochastic and selective processes can affect the deployment of intra-individual clonal diversity.

**Extended Data Fig. 1:**
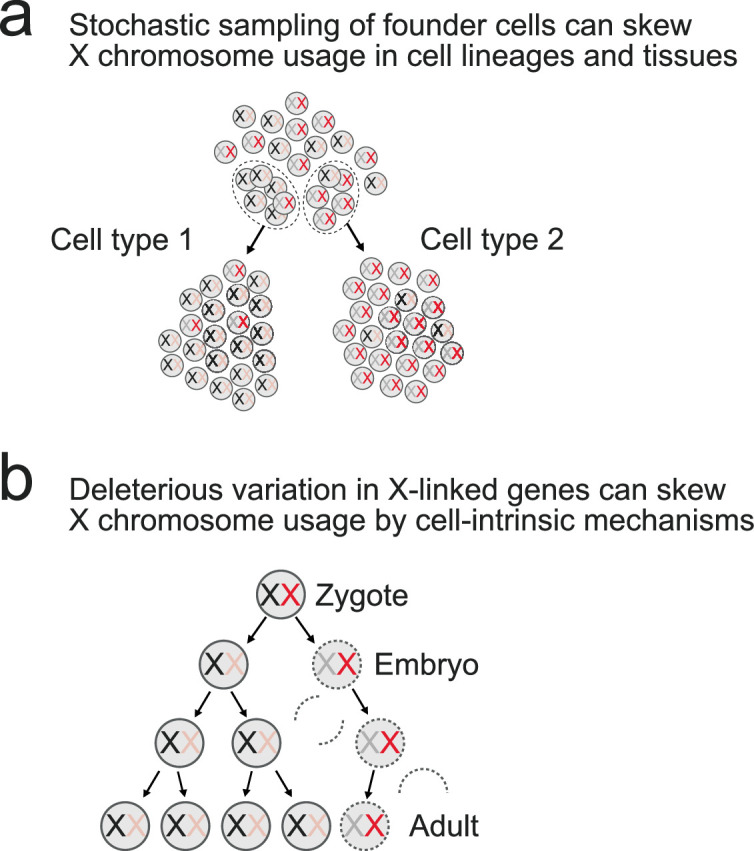
Stochastic and cell-intrinsic mechanisms can affect the representation of X-linked variation. **a**, Stochastic sampling of founder cells can result in skewed X chromosome usage that varies between cell lineages and tissues. **b**, Deleterious variation in X-linked genes can skew X chromosome usage by cell-intrinsic failure of clones to expand or survive.

Stochastic X-linked bias can arise from sampling errors early when founder cells are allocated to the three germ layers (ectoderm, endoderm and mesoderm) in embryonic development and can be further amplified by the allocation of cells to particular fates within each germ layer^4^ (Extended Data Fig. 1a). The resulting bias has been exploited to estimate the number of founder cells for cell types and tissues in embryonic development^4^ and the number of hematopoietic stem cells (HSCs) that contribute to the regeneration of blood cells in later life^11^.

A distinct form of X-linked bias arises from clonal selection against deleterious genetic variants that compromise the ability of variant-expressing clones to expand or survive in a cell-intrinsic fashion (Extended Data Fig. 1b). Clonal selection results in the dominance of clones that have inactivated the X chromosome harboring the deleterious variant and is relevant in the context of human disease, where intra-individual clonal diversity can mean a more favorable outcome in XX than XY individuals^2,12^.

Here we ask a different question, namely whether epigenetically diverse clones, which arise from the combined effect of XCI and X-linked genetic variation, merely co-exist in XX individuals, or whether they interact, and, if so, how such interactions may shape the landscape of X-linked clonal diversity. To this end, we generate mouse models of X-linked genetic variation found in the human *STAG2* gene and uncover a noncell-autonomous mode of X-linked bias which is distinct from stochastic variation and selection against deleterious variants. We find that clones expressing *Stag2* variants fail to adopt a lymphoid fate in the presence of competitor clones that have silenced the variant allele by XCI. Unexpectedly, however, the absence of competitors expressing wild-type (WT) *Stag2* restored the full range of cell fate choices to clones expressing *Stag2* variants. Our observations reveal that clonal interactions have the potential to shape the contribution of X-linked genetic diversity to specific cell types and tissues in XX individuals.

## Results

### Sequence variation and XCI combine to generate intra-individual genetic diversity

Analysis of 3,775 X chromosomes across 2,504 individuals from phase 3 of the 1000 Genomes Project^13^ found 13,796 nonsynonymous SNPs (SNPs that alter the amino acid sequence of proteins encoded on the X chromosome). The average number of such missense variants between any two X chromosomes was 138 (minimum = 3 and maximum = 232), omitting genes that escape X-inactivation in humans^3,4^. Ninety percent of X chromosome pairs harbored at least 101 missense variants. This analysis shows that sequence variation has the potential to generate intra-individual diversity in XX individuals when combined with XCI and its clonal propagation (Fig. 1a,b).

### Sequence variants in the X-linked *STAG2* gene disrupt cohesin–CTCF binding

**Extended Data Fig. 2:**
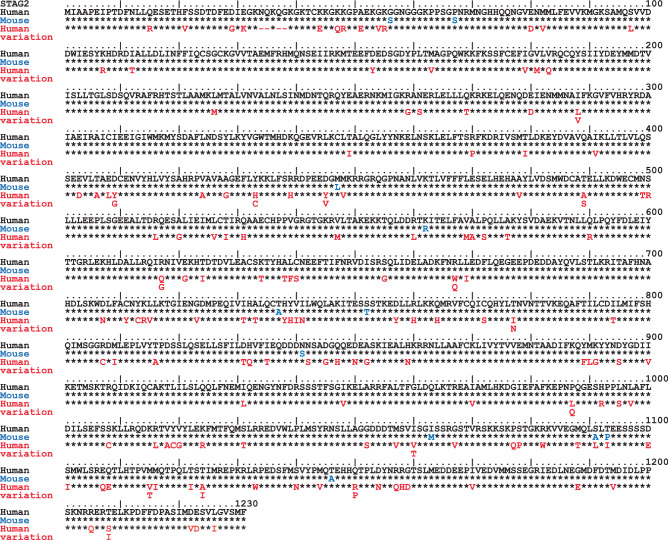
Sequence variation of the X-linked *STAG2* gene. STAG2 protein sequence alignment of human (black, sp|Q8N3U4|STAG2_HUMAN), mouse (blue, sp|O35638|STAG2_MOUSE) and sequence variation in the human population (red, gnomAD v2.1.1), excluding disease-associated variants from ClinVar or other patient databases. Alignment was performed by CLUSTAL O (1.2.4) based on Uniprot STAG2 ENSEMBL transcript ENST000003218089.9 (1231aa). gnomAD variant X-123185062—G-C (GRCh37) is highlighted. The variant has a site quality value = 3.46e+2 and genotype quality of 95–100% (allele number = 164895, allele frequency = 0.0000061 and changes STAG2 arginine 370 to proline (R370P) on one X chromosome in an XX individual).

*STAG2* is an essential X-linked gene that is evolutionarily highly conserved^14^ (Fig. 1c and Extended Data Fig. 2) and encodes a subunit of cohesin, a protein complex that contributes to 3D genome organization as well as DNA replication, DNA repair and the stable propagation of chromosomes through cell division^15^. A survey of 125,748 human exomes^10^ (gnomAD v2.1) found that *STAG2* coding variation was lower than predicted by chance, indicating a level of constraint expected for an essential gene (Fig. 1c,d). Nevertheless, >150 distinct missense variants were observed (Fig. 1c and Extended Data Fig. 2). We focused on gnomAD variant X-123185062—G-C (GRCh37) found in HG02885, an XX individual of African origin who self-reported as healthy, and participated with her husband and daughter in the control (nondisease) cohort of gnomAD v2.1.1. This SNP changes STAG2 arginine 370 to proline (R370P). STAG2 R370 contributes to an interaction interface that is formed jointly by the cohesin subunits STAG1/STAG2 and RAD21 (Fig. 1e). This interface has been described as a ‘conserved essential surface’ and is bound by the following cohesin-interacting proteins that are engaged in a range of DNA-based processes: CTCF in 3D genome organization^16^ (Fig. 1e), Shugoshin in sister chromatid cohesion^17,18^, MCM3 (minichromosome maintenance protein 3) in DNA replication^19^ and likely other cohesin interaction partners^20^. We used isothermal calorimetry to assess the impact of STAG2^R370P^ on cohesin–CTCF interactions and found a complete loss of binding (Fig. 1f). Hence, sequence variation in the X-linked *STAG2* gene illustrates the potential for clonal heterogeneity within XX individuals.

### *Stag2*^variant^ progenitors fail to form lymphocytes in heterozygous XX individuals

**Extended Data Fig. 3:**
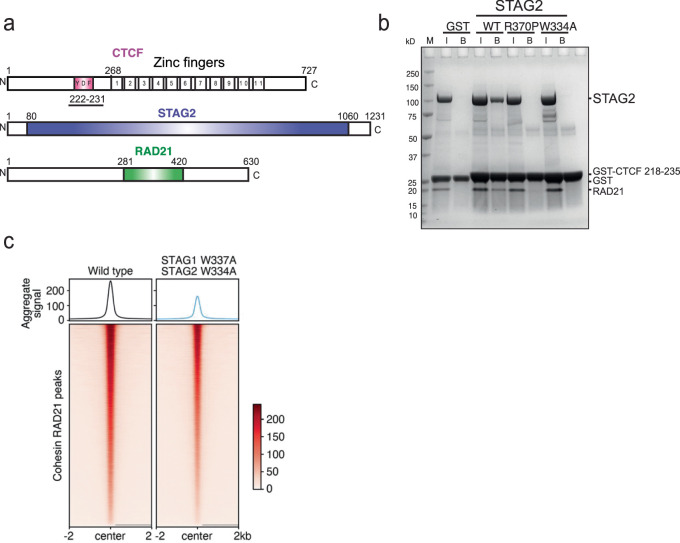
Characterization of STAG2 variants. **a**, Schematic of CTCF, STAG2 and RAD21. The regions of each protein used for in vitro binding assays are highlighted. **b**, Characterization of protein preparations used for isothermal calorimetry experiments. GST-CTCF pull-down. I, input; B, bound fraction; M, molecular weight marker. **c**, Variants in the conserved essential surface form chromatin-associated cohesin complexes. Cohesin complex formation and chromatin association of variants were tested by chromatin immunoprecipitation of the cohesin subunit RAD21 in HAP1 cells. Both *STAG2* W334A and *STAG1* W337A were mutated to rule out complementation of variant *STAG2* by WT *STAG1*. Note that a moderate reduction in the association of cohesin with chromatin is expected in *STAG1*^W337A^ *STAG2*^W334A^ cells, as cohesin is no longer stabilized by CTCF interactions^16^.

To explore the impact of X-linked sequence variation at the organismal level, we generated mouse models of *Stag2* variants in the conserved essential surface between STAG2 and CTCF (Fig. 1e). *Stag2*^R370Q^ had a tenfold lower CTCF binding affinity than WT (Fig. 1f). A second variant, *Stag2*^W334A^, abolished the STAG2–CTCF interaction to the same extent as the human R370T variant (Fig. 1f). As expected^16^, STAG2–CTCF interface variants retained the ability to form DNA-bound cohesin complexes (Extended Data Fig. 3c). *Stag2*^R370Q^ and *Stag2*^W334A^ variants showed equivalent phenotypes and are therefore described together.

**Fig. 2:**
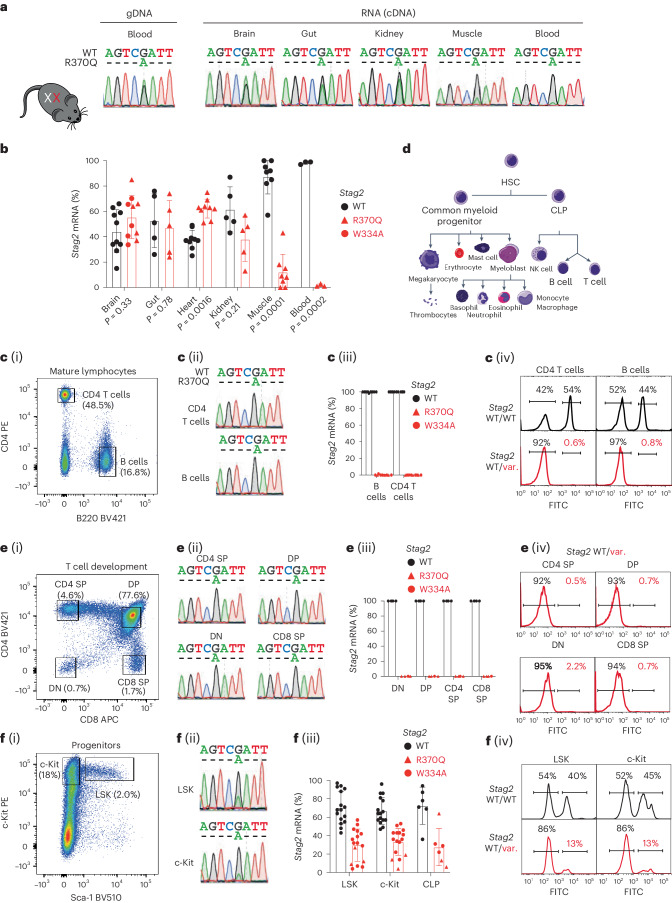
*Stag2*^variant^ clones fail to form lymphocytes in *Stag2*^WT^*Stag2*^variant^ individuals. **a**, Sanger sequencing of *Stag2*^WT^ and *Stag2*^variant^ gDNA (top) and cDNA (bottom) as an indicator for the representation of *Stag2*^WT^ and *Stag2*^variant^ clones in tissues from heterozygous females. Muscle, skeletal muscle. Blood, blood mononuclear cells. **b**, Allele-specific qRT–PCR as a quantitative assay for the representation of *Stag2*^WT^ and *Stag2*^variant^ clones in tissues from heterozygous females. Mean ± s.d. of three to eight biological replicates. Gut, small intestine. *P* values, one-sample *t* test comparing the observed mean to the expected (50%). **c** (i), Mature CD4 T and B lymphocytes from *Stag2*^WT^ *Stag2*^variant^ heterozygous females. (ii) Sanger sequencing of cDNA. (iii) Allele-specific qRT–PCR (*n* = 9). (iv) Live-cell reporter assay for the representation of *Stag2*^WT^ (FITC-negative) and *Stag2*^variant^ *Atrx*^*Luc*/*βGal*^ (FITC-positive) clones in mature CD4 T cells (*n* = 6, mean = 1.7 ± 1.7% FITC-positive) and B cells (*n* = 6, mean = 3.3 ± 3.2% FITC-positive) at the single-cell level (red histogram). X^*Stag2-*WT^ and X^*Stag2-*WT *Atrx-Luc*/*βGal*^ heterozygous cells are shown as control (black histogram). **d**, Schematic representation of hematopoiesis, modified from https://commons.wikimedia.org/w/index.php?curid=7351905. **e** (i), Thymocyte populations at consecutive developmental stages—CD4/CD8 DN, DP, CD4 or CD8 single positive (SP) of *Stag2*^WT^ *Stag2*^variant^ heterozygous females. (ii) Sanger sequencing of *Stag2*^WT^ *Stag2*^variant^ thymocyte cDNA. (iii) Allele-specific qRT–PCR of *Stag2*^WT^ and *Stag2*^variant^ thymocyte cDNA (*n* = 4). (iv) Live-cell reporter assay for the representation of *Stag2*^WT^ (FITC-negative) and *Stag2*^variant^ (FITC-positive) clones in thymocyte subsets. Genotypes as in **c**. **f** (i), Bone marrow stem (LSK) and progenitor (c-kit) cells from *Stag2*^WT^ *Stag2*^variant^ heterozygous females. (ii) Sanger sequencing of hematopoietic stem and progenitor cell cDNA. (iii) Allele-specific qRT–PCR for the representation of *Stag2*^variant^ and *Stag2*^WT^ clones in hematopoietic stem and progenitor cells mean ± s.d. of 5–15 biological replicates. One-sample *t* test comparing the mean of *Stag2*^variant^ to the expected mean of 50% (LSK and c-kit, *P* = 0.0003; Lin^−^ c-kit^+^ FLT3^+^ CD127^+^ CLP, *P* = 0.04). (iv) Live-cell reporter assay for the representation of *Stag2*^WT^ (FITC-negative) and *Stag2*^variant^ (FITC-positive) clones in hematopoietic stem and progenitor cells. LSK *(n* = 7, mean = 16.8 ± 9.7% FITC-positive), c-kit (*n* = 7, mean = 17.5 ± 10.0% FITC-positive) and CLP *(n* = 3, mean = 14.1 ± 12.8% FITC-positive). Genotypes as in **c**. NK cell, natural killer cell. Source data

**Extended Data Fig. 4:**
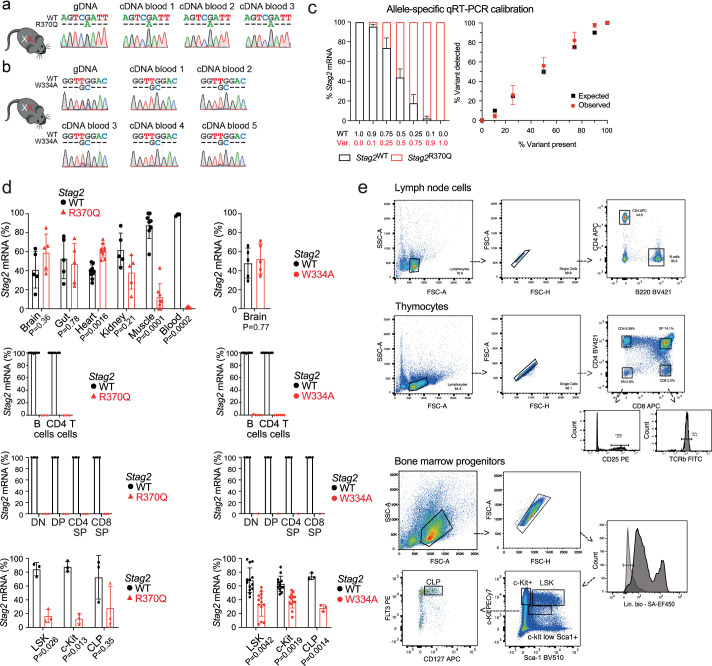
Representation of *Stag2*^variant^ clones in heterozygous XX individuals. **a**, Analysis of the representation of clones with active *Stag2*^wild-type^ versus *Stag2*^variant^ in blood mononuclear cells by Sanger sequencing in females heterozygous for *Stag2*^R370Q^. **b**, Analysis as in **a**, but for *Stag2*^W334A^. **c**, Calibration of allele-specific qRT–PCR of *Stag2*^wild-type^ and *Stag2*^variant^ cDNA. Left: ratio of *Stag2*^wild-type^ and *Stag2*^variant^ mRNA (y-axis) extracted from bone marrow progenitors containing the indicated proportions of cells (x-axis). Right: comparison of expected vs observed *Stag2*^*v*ariant^ mRNA ratios. Mean ± SD of 2 biological replicates. **d**, Allele-specific qRT-PCR for *Stag2*^WT^ and *Stag2*^variant^ clones in heterozygous females as shown in Fig. 2, with the exception that data for *Stag2*^R370Q^ and *Stag2*^W334A^ are displayed separately. **e**, Gating strategies used to isolate lymph node cells, thymocytes, and bone marrow progenitors. Expression of lineage markers on bone marrow cells (Lin SA-EF450) is shown before (dark gray) and after depletion with streptavidin beads (light gray). Source data

WT and variant *Stag2* were equally represented in genomic DNA (gDNA) from heterozygous X^*Stag2-*WT^ and X^*Stag2-*variant^ female mice, as illustrated for gDNA from blood (Fig. 2a, left). An equivalent representation of *Stag2*^WT^ and *Stag2*^variant^ genomic sequences was expected, as the presence of gDNA is unaffected by the epigenetic inactivation of one X chromosome in XX individuals^1^. We next analyzed a range of cell types and tissues in heterozygous female mice to determine the contribution of clones in which the active X chromosome harbored the *Stag2*^WT^ allele (*Stag2*^WT^ clones) versus clones in which the active X chromosome harbored the *Stag2*^variant^ allele (*Stag2*^variant^ clones). We isolated RNA, reverse-transcribed RNA into cDNA and sequenced the complementary DNA (cDNA). Brain, gut and other tissues showed a roughly equal representation of *Stag2*^WT^ and *Stag2*^variant^ clones (Fig. 2a), while skewing toward *Stag2*^WT^ clones was found in skeletal muscle (Fig. 2a). cDNA isolated from peripheral blood mononuclear cells showed a markedly reduced expression of variant *Stag2* (Fig. 2a and Extended Data Fig. 4a,b), indicating a near-complete absence of *Stag2*^variant^ clones.

To quantify the contribution of *Stag2*^variant^ versus *Stag2*^WT^ clones, we used allele-specific qRT–PCR (see Extended Data Fig. 4c for calibration). This analysis confirmed reduced representation of *Stag2*^variant^ clones in blood mononuclear cells (Fig. 2b) and in skeletal muscle and revealed increased representation of *Stag2*^variant^ clones in the heart (Fig. 2b and variants are shown separately in Extended Data Fig. 4d).

**Extended Data Fig. 5:**
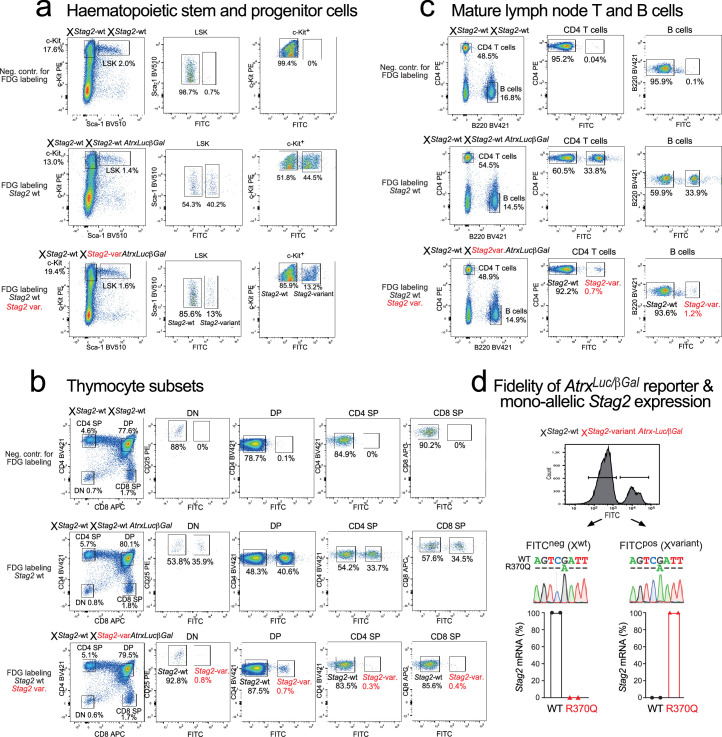
Live-cell reporter assay for the representation of *Stag2*^wild-type^ and *Stag2*^variant^ clones among hematopoietic cell populations at the single-cell level. **a**, Hematopoietic stem and progenitor cells. Live-cell reporter assay for the representation of *Stag2*^wild-type^ (FITC-negative) and *STAG2*^variant^ (FITC-positive) clones in hematopoietic stem (LSK) and progenitor (c-kit) cells from bone marrow. See **b** for details. **b**, Thymocyte subsets. Live-cell reporter assay for the representation of *Stag2*^wild-type^ (FITC-negative) and *STAG2*^variant^ (FITC-positive) clones in thymocyte subsets. See **b** for details. **c**, Mature lymph node T and B cells. Live-cell reporter assay for the representation of *Stag2*^wild-type^ (FITC-negative) and *STAG2*^variant^ (FITC-positive) clones in lymph node CD4 T and B cells. Top: *Stag2*^wild-type^ female lacking the *Atrx*^*Luc*/*βGal*^ reporter. Middle: *Stag2*^wild-type^ female heterozygous for *Atrx*^*Luc/βGal*^. Bottom: heterozygous *Stag2*^wild-type^ *Stag2*^variant^ female with *Atrx*^*Luc*/*βGal*^ reporter allele located on the same X chromosome as the *STAG2*^variant^ allele. **d**, Fidelity of the *Atrx*^*Luc*/*βGal*^ reporter allele and selective expression of *Stag2* alleles. Lineage-negative BM cells from female mice that were heterozygous for the Stag2 variant R370Q and the *Atrx*^*Luc*/*βGal*^ reporter allele on the same chromosome were labeled with FDG and sorted into FITC-negative and FITC-positive cells by flow cytometry. Sanger sequencing of cDNA and allele-specific qRT–PCR were performed to determine the expression of wild-type and variant *Stag2*. Note that FITC-negative cells expressed exclusively *Stag2*^wild-type^, and FITC-positive cells expressed exclusively *Stag2*^variant^. Two independent biological replicates.

T and B lymphocytes are the major mononuclear cell types in blood. CD4 T and B cells isolated from lymph nodes of *Stag2*^variant^ *Stag2*^WT^ heterozygous females (Fig. 2c(i) and gating strategy in Extended Data Fig. 4e) showed a near-complete absence of *Stag2*^variant^ clones as determined by sequencing (Fig. 2c(ii)) and allele-specific qRT–PCR (Fig. 2c(iii)). We developed a reporter system to directly visualize individual cells expressing *Stag2*^variant^ or *Stag2*^WT^ by inserting a *Luc*/*βGal* reporter construct^21,22^ into the X-linked *Atrx* gene, which is subject to XCI and broadly expressed across cell types and tissues, including the hematopoietic system^23^. *Atrx*^*Luc*/*βGal*^ allows the visualization and prospective isolation of live *Atrx*^*Luc*/*βGal*^ cells by flow cytometry, based on the conversion of nonfluorescent fluorescein di-β-d-galactopyranoside (FDG) into green fluorescent fluorescein isothiocyanate (FITC) by the enzymatic activity of β-galactosidase (βGal). We confirmed that FDG conversion was indeed dependent on the presence of the *Atrx*^*Luc*/*βGal*^ reporter (Extended Data Fig. 5a–c). In female mice that were heterozygous for the *Atrx*^*Luc*/*βGal*^ reporter and had two WT alleles of *Stag2*, FDG to FITC conversion occurred in approximately half of all T and B lymphocytes (Fig. 2c(iv), top) and other hematopoietic cell types examined (Extended Data Fig. 5a–c). This indicates that the reporter itself does not substantially skew X chromosome usage. Sanger sequencing and allele-specific qRT–PCR confirmed the fidelity of the reporter, as well as the monoallelic expression of *Stag2* in XX individuals (Extended Data Fig. 5d). In lymphocytes isolated from *Stag2*^WT^ *Stag2*^variant^ *Atrx*^*Luc*/*βGal*^ heterozygous females, *Stag2*^WT^ clones dominated over *Stag2*^variant^ *Atrx*^*Luc*/*βGal*^ clones (Fig. 2c(iv), bottom, and Extended Data Fig. 5c). Taken together with the sequencing and allele-specific qRT–PCR data, these results indicate that *Stag2*^variant^ clones fail to contribute substantially to mature T and B lymphocytes in *Stag2*^WT^ *Stag2*^variant^ heterozygous females.

**Extended Data Fig. 6:**
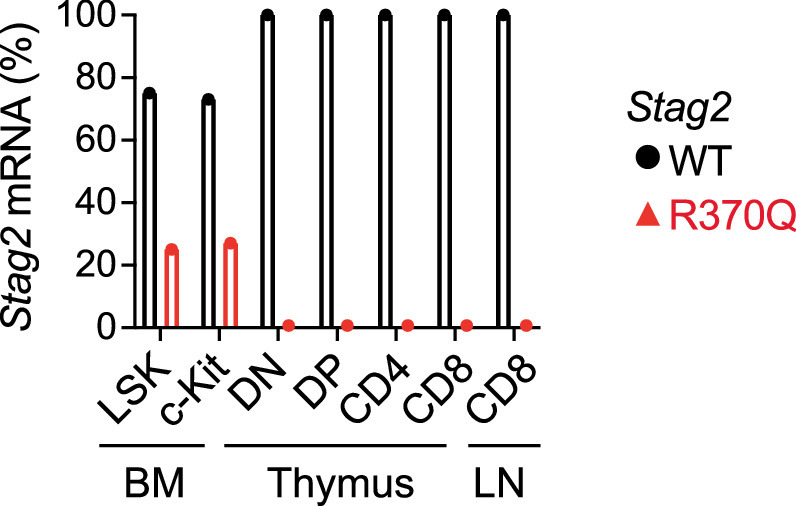
Rearranged T cell receptor transgenes fail to rescue the differentiation of *Stag2* variant progenitor cells. Allele-specific qRT–PCR of *Stag2*^wild-type^ and *Stag2*^variant^ cDNA isolated from bone marrow progenitors (BM), thymocyte subsets (thymus) and CD8 lymph node T cells (LN) isolated from a heterozygous *Stag2*^wild-type^ *Stag2*^variant^ female harboring an OT-I T cell receptor transgene. One replicate (see Extended Data Fig. 4e for gating strategy). Source data

**Extended Data Fig. 7:**
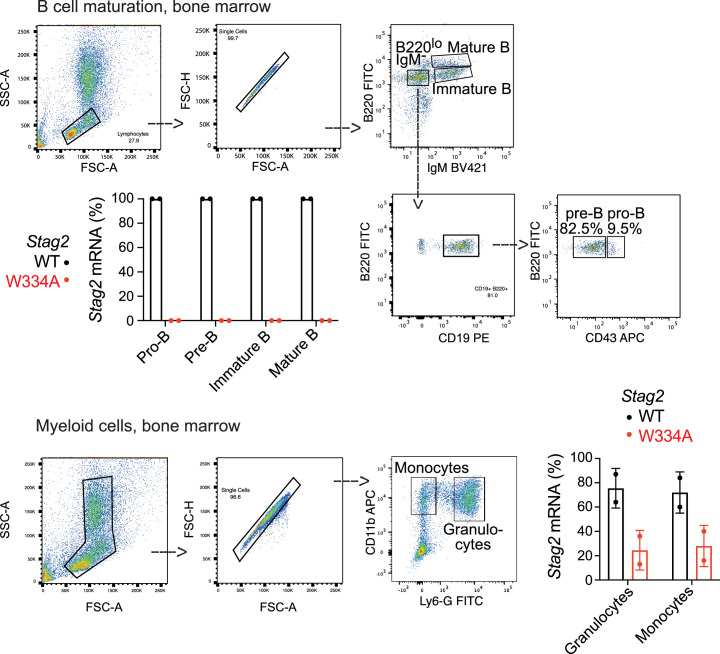
B cell development and myeloid cells in the bone marrow. Allele-specific qRT–PCR of *Stag2*^wild-type^ and *Stag2*^variant^ cDNA isolated from the indicated subsets of B cells (top) and myeloid cells (bottom) isolated from *Stag2*^wild-type^ *Stag2*^W334A^ heterozygous females. Mean ± SD of 2 biological replicates per population and genotype. B cell progenitors were defined as follows: pro-B (B220^lo^ CD19^+^ IgM^−^ CD43^+^), pre-B (B220^lo^ CD19^+^ IgM^−^ CD43^−^) and immature B (B220^lo^ CD19^+^ IgM^+^). Granulocytes (Cd11b^+^ Ly6-G^+^). Monocytes (Cd11b^+^ Ly6-G^−^). Source data

Blood cells are continuously replenished by hematopoietic stem and progenitor cells^11^ (Fig. 2d), allowing the developmental origin of skewed X chromosome usage to be traced. T cell fate specification of bone marrow-derived progenitors occurs in the thymus, and we, therefore, examined the representation of *Stag2*^variant^ clones among thymocyte subsets at successive stages of development (Fig. 2e(i) and gating strategy in Extended Data Fig. 4e). Sequencing (Fig. 2e(ii)), allele-specific qRT–PCR (Fig. 2e(iii)) and FDG labeling of *Stag2*^WT^ *Stag2*^variant^ *Atrx*^*Luc*/*βGal*^ thymocytes (Fig. 2e(iv) and Extended Data Fig. 5c) showed that *Stag2*^variant^ clones were barely detectable among developing T cells. Thymocyte differentiation of *Stag2*^variant^ clones was not rescued by provision of rearranged lymphocyte receptor transgenes (Extended Data Fig. 6). *Stag2*^variant^ clones were also absent from developing pro-B and pre-B cells in the bone marrow (Extended Data Fig. 7).

We next examined the representation of variant *Stag2* RNA in hematopoietic stem (LSK), c-kit^+^ and common lymphoid progenitor (CLP) cells isolated from the bone marrow of heterozygous *Stag2*^WT^ *Stag2*^variant^ female mice (Fig. 2f(i) and gating strategy in Extended Data Fig. 4e). Sequencing (Fig. 2f(ii)), allele-specific qRT–PCR (Fig. 2f(iii)) and FDG labeling (Fig. 2f(iv) and Extended Data Fig. 5b) revealed skewing against *Stag2*^variant^ clones in hematopoietic stem and progenitor cells. In contrast to lymphocytes, the representation of *Stag2*^variant^ clones among mature myeloid cells remained comparable to hematopoietic stem and progenitor cells (Extended Data Fig. 7).

In conclusion, the hematopoietic system of *Stag2*^WT^ *Stag2*^variant^ heterozygous individuals appeared outwardly normal with respect to the number and composition of cell types in bone marrow, thymus and peripheral lymph nodes. However, the clonal composition of the hematopoietic system was skewed toward *Stag2*^WT^ clones, and few, if any, *Stag2*^variant^ clones contributed to immature and mature lymphocyte subsets. These findings suggested that hematopoietic progenitors with an active X chromosome harboring *Stag2* variants were unable to undergo lymphoid specification and differentiation.

### Reduced lymphoid priming in *Stag2*^variant^ hematopoietic progenitors

**Fig. 3:**
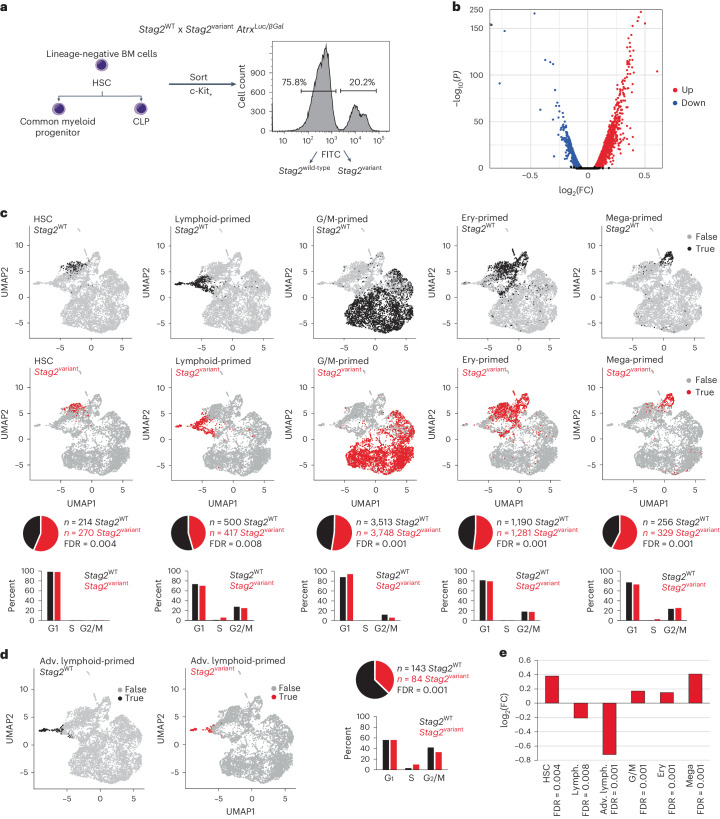
Reduced efficiency of lymphoid priming in *Stag2*^variant^ hematopoietic progenitors. **a**, Isolation of c-kit^+^ lineage-negative bone marrow cells by flow cytometry from heterozygous females that harbor the *Stag2*^R370Q^ variant and the *Atrx*^*Luc*/*βGal*^ reporter gene on the same X chromosome (see Extended Data Fig. 8 for details). **b**, Volcano plot of gene expression in merged multipotent and lineage-restricted hematopoietic progenitor cells (see Supplementary Data 1 for marker genes of multipotent and lineage-restricted progenitors and Supplementary Data 2 for differentially expressed genes). Differential expression analysis was conducted using a two-tailed Wald test, and *P* values were adjusted by the Benjamini–Hochberg correction implemented in DESeq2. **c**, Two-dimensional UMAPs. scRNA-seq data generated from sorted *Stag2*^WT^ and *Stag2*^variant^ c-kit^+^ lineage-negative bone marrow cells were analyzed for gene expression profiles corresponding to long-term HSC, lymphoid-primed, G/M-primed, Ery-primed and Mega-primed progenitors (see Supplementary Data 4 for marker genes of lineage priming). Each subset was analyzed for the expression of cell cycle markers to infer the cell cycle stage as indicated by histograms. The numbers for each subset are shown. Pie charts show the proportions of *Stag2*^WT^ and *Stag2*^variant^ progenitors for each subset normalized to the number of progenitors that passed QC metrics (*n* = 6,274 *Stag2*^WT^ and *n* = 6,073 *Stag2*^variant^). False discovery rates (FDR) were determined by permutation test. **d**, UMAPs, numbers, proportions and cell cycle status of *Stag2*^WT^ and *Stag2*^variant^ advanced lymphoid-primed progenitors. FDR was determined by the permutation test. **e**, Summary of log_2_(FC) in the proportions of *Stag2*^variant^ progenitors of the indicated types. FDRs were determined by the permutation test. FC, fold change. Source data

**Extended Data Fig. 8:**
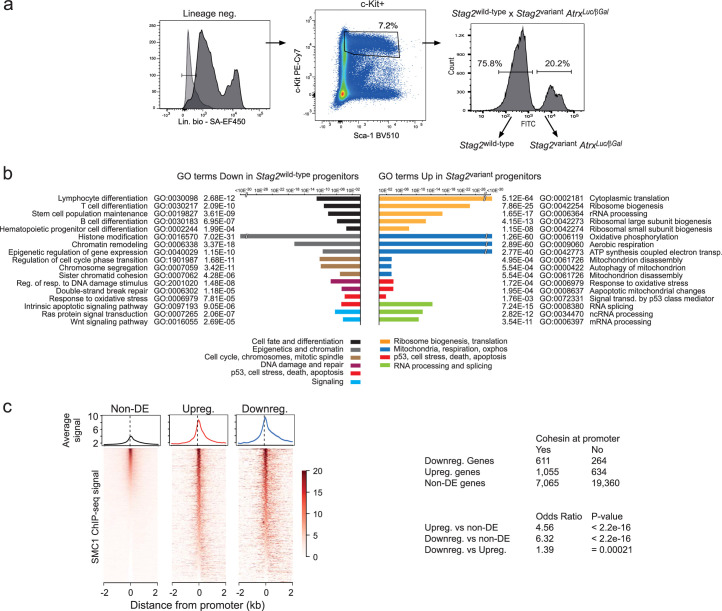
Isolation of *Stag2*^wild-type^ and *Stag2*^variant^ hematopoietic progenitors and analysis of differentially expressed genes. **a**, Isolation of lineage-negative c-kit^+^ bone marrow progenitors (see Extended Data Fig. 4d for the gating strategy). Lineage markers are shown before and after depletion of lineage-positive cells. FDG staining of lineage-negative bone marrow cells from heterozygous females that harbor *Stag2*^wild-type^ on one X chromosome and the *Stag2*^R370Q^ on the other, along with the *Atrx*^*Luc/βGal*^ reporter. **b**, Representative gene ontology terms ‘biological function’ of genes found upregulated (right) or downregulated (left) in *Stag2*^R370Q^ versus *Stag2*^wild-type^ hematopoietic progenitor cells isolated from *Stag2*^wild-type^ *Stag2*^R370Q^ *Atrx*^*Luc/βGal*^ mice. The horizontal axis displays Benjamini–Hochberg adjusted P-values and is truncated at *P* < 10E−30. Significance was determined by one-sided Fisher's exact test implemented in ClusterProfiler (see Supplementary Data 3 for a full list of GO terms). **c**, Heatmaps of cohesin binding at gene promoters in hematopoietic progenitor cells. Promoters were classified as upregulated, downregulated or not deregulated according to the status of the associated transcripts in *Stag2*^variant^ versus *Stag2*^wild-type^ progenitors. Odds ratios and P-values were calculated by two-sided Fisher’s exact test. The nominal values for P-values given as P < 2.2 10e-16 are 1.05e-192 for upregulated vs non-DE and 1.73e-149 for downregulated vs non-DE.

We isolated lineage-negative, c-kit^+^ *Stag2*^WT^ and *Stag2*^variant^ cells from the bone marrow of heterozygous females for single-cell RNA-sequencing (scRNA-seq; Fig. 3a, Extended Data Fig. 8a and gating strategy in Extended Data Fig. 4e) and identified progenitors based on established marker genes (Supplementary Data 1). DESeq2 found 1,600 upregulated and 802 downregulated genes in *Stag2*^variant^ progenitors (adjusted *P* < 0.01; Fig. 3b and representative gene ontology terms in Extended Data Fig. 8b). As STAG2 is part of the cohesin complex, we analyzed the relationship between cohesin binding and deregulated gene expression in *Stag2*^variant^ progenitors. Leveraging cohesin chromatin immunoprecipitation followed by sequencing (ChIP–seq) from hematopoietic progenitors, we found that genes that were deregulated in *Stag2*^variant^ progenitors were highly enriched for cohesin promoter binding compared to non-deregulated genes (Extended Data Fig. 8c), which links transcriptional deregulation in *Stag2*^variant^ cells to cohesin.

We harnessed scRNA-seq gene expression profiles to identify long-term HSCs and lineage-primed progenitors among *Stag2*^variant^ and *Stag2*^WT^ progenitors. While the absolute number of *Stag2*^variant^ progenitors was reduced compared to *Stag2*^WT^, the progenitors that were present in *Stag2*^variant^ showed an increased proportion of HSCs relative to *Stag2*^WT^ (Fig. 3c). Analysis of cell cycle markers suggested that *Stag2*^WT^ and *Stag2*^variant^ HSCs were largely quiescent (~99% G1), while lineage-primed progenitors were cycling in both *Stag2*^WT^ and *Stag2*^variant^ (Fig. 3c). The proportion of *Stag2*^variant^ lymphoid-primed progenitors was reduced, while the proportions of granulocyte/macrophage (G/M)-primed, erythroid (Ery)-primed and megakaryocyte (Mega)-primed progenitors were increased among *Stag2*^variant^ progenitors (Fig. 3c). Reduced lymphoid priming of *Stag2*^variant^ progenitors was progressive, as indicated by a further reduction in the proportion of *Stag2*^variant^ advanced lymphoid-primed progenitors that expressed a greater number of lymphoid genes (AUCell score of ≥0.2; Fig. 3d), although cell cycle profiles of lymphoid-primed progenitors were comparable between *Stag2*^WT^ and *Stag2*^variant^ progenitors (Fig. 3c,d). Figure 3e summarizes log_2_(fold change) in the proportions of *Stag2*^variant^ progenitor subsets. Hence, despite the failure of *Stag2*^variant^ hematopoietic progenitors to form early B and T cells (pro-B cells and double-negative (DN) thymocytes, respectively), scRNA-seq provided evidence of lymphoid priming, albeit with reduced efficiency compared to *Stag2*^WT^ progenitors.

### Competition between *Stag2*^variant^ and *Stag2*^WT^ clones

**Fig. 4:**
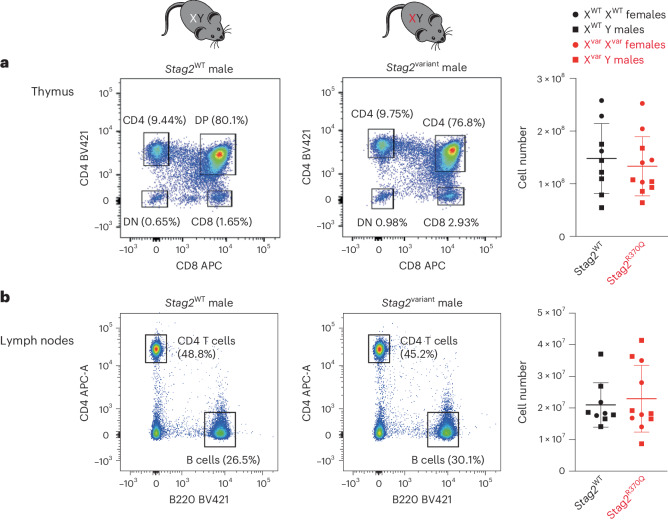
Successful lymphoid specification and differentiation of *Stag2*^variant^ cells in the absence of *Stag2*^WT^. **a**, Flow cytometry of thymocyte subsets (left) and thymus cell numbers (right) in control versus hemizygous *Stag2*^variant^ males and homozygous *Stag2*^variant^ females. Mean ± s.d. of 9–11 biological replicates (unpaired *t* test, *P* = 0.59). **b**, Flow cytometry of lymph node cells (left) and lymph node cell numbers (right) in control versus hemizygous *Stag2*^variant^ males and homozygous *Stag2*^variant^ females. Mean ± s.d. of 9–11 biological replicates (unpaired *t* test, *P* = 0.64). Source data

Based on these results, we wondered whether the failure of clones expressing variant *Stag2* to contribute to lymphoid lineages was entirely due to cell-intrinsic defects that preclude lymphoid cell fate specification. To address this question, we generated *Stag2*^variant^ hemizygous males and *Stag2*^variant^ homozygous females, which exclusively harbored *Stag2*^variant^ cells. To our surprise, we found that in the absence of *Stag2*^WT^, the cellularity and subset distribution of *Stag2*^variant^ thymocytes (Fig. 4a) and lymph node cells (Fig. 4b) were indistinguishable from WT controls in *Stag2*^variant^ hemizygous males and *Stag2*^variant^ homozygous females.

**Extended Data Fig. 9:**
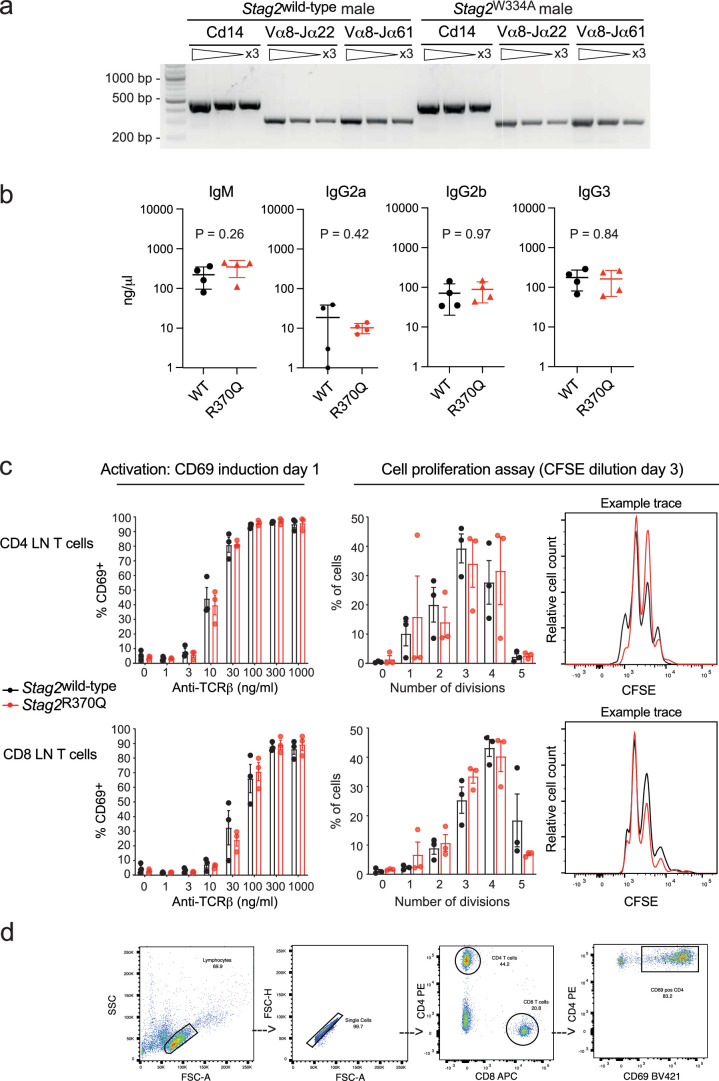
*Stag2*^variant^ lymphocytes are competent to undergo secondary *Tcra* rearrangements, *Igh* class switch recombination and *in vitro* activation. **a**, Threefold dilutions of genomic Vα8-Jα PCR products obtained from DP thymocytes sorted from *Stag2*^variant^ compared to *Stag2*^wild-type^ males. *Cd14* was used as a genomic control. One of three similar biological replicates. **b**, Concentrations of the indicated immunoglobulin isotypes were determined by enzyme-linked immunosorbent assay in the sera of unimmunized adult *Stag2*^wild-type^ and *Stag2*^variant^ males. Four independent biological replicates were analyzed per genotype. P-values were determined by unpaired two-tailed t-test. **c**, Lymph node cells were activated using plate-bound H57 anti-TCRβ antibodies at the indicated concentrations, together with 2 μg/ml soluble anti-CD28. Left: CD69 expression was assessed by flow cytometry after 1 day of activation. Middle: the fraction of cells that completed the indicated number of cell divisions as determined by flow cytometric assessment of CFSE dilution. Right: representative CFSE traces. Mean ± SEM of 3 biological replicates per genotype. **d**, Gating strategy used in **c**. Source data

Cohesin is required for secondary rearrangements at the *Tcra* locus in immature thymocytes^24^ and class switch recombination at the *Igh* immunoglobulin heavy chain locus in B cells^25,26^. Unlike *Rad21*^ko^ thymocytes, *Stag2*^variant^ thymocytes rearrange both proximal (Jα61) and distal (Jα22) *Tcra* gene segments to a similar extent as *Stag2*^WT^ thymocytes (Extended Data Fig. 9a). Similarly, we found WT concentrations of immunoglobulin isotypes in *Stag2*^variant^ mice, indicating class switch recombination (Extended Data Fig. 9b). Mature lymphocytes are quiescent, but upon engagement of their receptors for antigen and costimulatory ligands, they undergo a program of activation that culminates in cell cycle entry and cellular proliferation. We activated T cells with antibodies to the T cell receptor at graded concentrations, together with a fixed dose of antibody to the costimulatory receptor CD28. As a readout, we measured the expression of the activation marker CD69 by flow cytometry (Extended Data Fig. 9c, left, and gating strategy in Extended Data Fig. 9d) and assessed T cell proliferation by carboxyfluorescein succinimidyl ester (CFSE), which fluorescently labels cellular proteins that are diluted twofold at each successive cell division (Extended Data Fig. 9c, middle and right). The results showed that *Stag2*^variant^ CD4 and CD8 T cells generated in X^*Stag2-*variant^ hemizygous males were as responsive to activation signals as *Stag2*^WT^ cells.

We conclude that *Stag2*^variant^ progenitors can generate lymphocytes that are competent to undergo *Tcra* rearrangement, *Igh* class switch recombination and in vitro activation. However, *Stag2*^variant^ progenitors fail to realize their lymphoid potential in the presence of *Stag2*^WT^ cells. The impact of *Stag2*^WT^ cells on *Stag2*^variant^ progenitors is reminiscent of a form of cell competition whereby cells are eliminated only when they differ from their neighbors^27–29^.

### *Stag2*^variant^ progenitors retain lymphoid potential in the face of competition

**Fig. 5:**
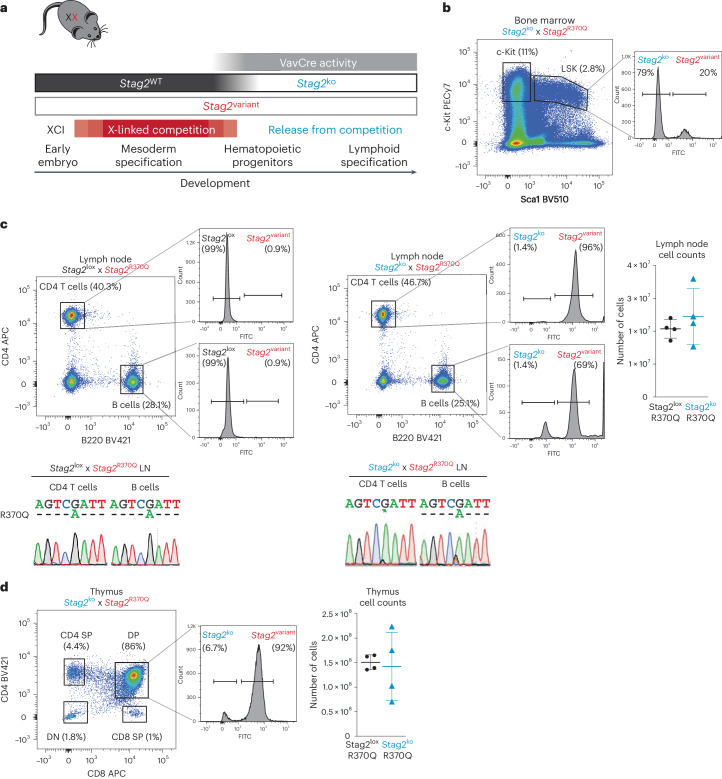
*Stag2*^variant^ progenitors in heterozygous females retain the potential to support lymphoid specification and differentiation. **a**, Outline of the experiment. Before VavCre activation, *Stag2*^*lox*^ is fully functional, and *Stag2*^variant^ clones therefore compete against *Stag2*^WT^ clones from the point of XCI in the early embryo up to entry into the hematopoietic progenitor cell pool. VavCre expression in hematopoietic progenitor cells converts *Stag2*^*lox*^ into *Stag2*^ko^, and *Stag2*^variant^ clones now compete with *Stag2*^ko^ instead of *Stag2*^WT^ clones. **b**, Analysis of hematopoietic progenitors in VavCre^+^ *Stag2*^ko^ *Stag2*^R370Q^ *Atrx*^*Luc*/*βGal*^ mice by flow cytometry. One experiment is representative of four independent biological replicates. Live-cell reporter assay for the representation of FITC^+^ *Stag2*^variant^ clones. **c**, Analysis of lymph node CD4 T and B cells populations isolated by flow cytometry from VavCre^−^ *Stag2*^lox^ *Stag2*^R370Q^ *Atrx*^*Luc*/*βGal*^ controls (left) and VavCre^+^ *Stag2*^ko^ *Stag2*^R370Q^ *Atrx*^*Luc*/*βGal*^ mice (right). One experiment is representative of four independent biological replicates. Cell numbers are shown on the right. Sanger sequencing of cDNA isolated from the indicated cell populations derived from VavCre^neg^ *Stag2*^lox^ *Stag2*^R370Q^ controls (left) and VavCre^pos^ *Stag2*^ko^ *Stag2*^R370Q^ mice (right). Live-cell reporter assay for the representation of FITC^+^ *Stag2*^variant^ clones in mature CD4 T and B cells at the single-cell level. **d**, Analysis of thymocyte populations in VavCre^+^ *Stag2*^ko^ *Stag2*^R370Q^ *Atrx*^*Luc*/*βGal*^ mice by flow cytometry. One experiment is representative of four independent biological replicates. Cell numbers are indicated on the right. Live-cell reporter assay for the representation of FITC^+^ *Stag2*^variant^ clones in immature (DP) thymocytes. Source data

As described above, *Stag2*^variant^ clones are detectable in the hematopoietic progenitor pool of heterozygous *Stag2*^variant^ *Stag2*^WT^ individuals and undergo at least limited lymphoid priming, but fail to substantially contribute to lymphoid specification and differentiation. Given that *Stag2*^variant^ clones were potentially exposed to competition throughout embryonic development, they may already be wounded or damaged beyond rescue by the time they enter the hematopoietic progenitor pool in heterozygous *Stag2*^variant^ *Stag2*^WT^ females. To gain additional insights into the rules of X-linked competition, we generated heterozygous *Stag2*^variant^ *Stag2*^*lox*^ female mice. The *Stag2*^*lox*^ allele encodes normal levels of WT STAG2 protein, but when deleted by Cre recombinase, it curtails differentiation of *Stag2*^ko^ progenitors into lymphocytes^30^. We used *VavCre*^31^ to delete *Stag2* upon entry into the hematopoietic progenitor pool (Fig. 5a). In this experimental setting, clones expressing variant *Stag2* face competition from *Stag2*^WT^ cells until *VavCre* expression in hematopoietic progenitors. *VavCre* converts *Stag2*^lox^ into *Stag2*^ko^, effectively releasing *Stag2*^variant^ progenitors from competition by *Stag2*^WT^ cells (Fig. 5a). We used the *Atrx*^*Luc*/*βGal*^ reporter integrated into the X chromosome harboring the *Stag2*^R370Q^ variant to determine the abundance of *Stag2*^variant^ clones. *Stag2*^variant^ clones continued to be outnumbered in the hematopoietic stem and progenitor compartment of VavCre^+^ *Stag2*^ko^ *Stag2*^R370Q^ bone marrow (Fig. 5b), as observed in *Stag2*^WT^ *Stag2*^variant^ mice. Lymph nodes of VavCre^pos^ *Stag2*^ko^ *Stag2*^R370Q^ heterozygous females showed similar cellularity as VavCre^neg^ *Stag2*^lox^ *Stag2*^R370Q^ (Fig. 5b). However, in stark contrast to control VavCre^neg^ *Stag2*^lox^ *Stag2*^R370Q^ lymph node CD4 T and B cells, Sanger sequencing and the *Atrx*^*Luc*/*βGal*^ reporter indicated dominance of *Stag2*^variant^ transcripts in cDNA of VavCre^+^ *Stag2*^ko^ *Stag2*^R370Q^ lymph node CD4 T and B cells (Fig. 5c) and thymocytes (Fig. 5d) following deletion of *Stag2* ^lox^ by VavCre. As expected, *Stag2*^variant^ clones generated few—if any—lymphocytes in VavCre^−^ *Stag2*^lox^ *Stag2*^R370Q^ mice (Fig. 5c), where they competed against clones expressing WT STAG2 protein encoded by *Stag2*^lox^. These data show that the removal of *Stag2*^WT^ competition at the hematopoietic progenitor stage is sufficient to reveal the lymphoid potential of *Stag2*^variant^ progenitor cells.

Hence, *Stag2*^variant^ cells are capable of generating normal numbers of lymphocytes, either in the complete absence of *Stag2*^WT^ (that is, in hemizygous *Stag2*^variant^ males or homozygous *Stag2*^variant^ females) or on release from competition by selective removal of *Stag2*^WT^ cells from the hematopoietic progenitor pool.

### X-linked competition in humans

**Fig. 6:**
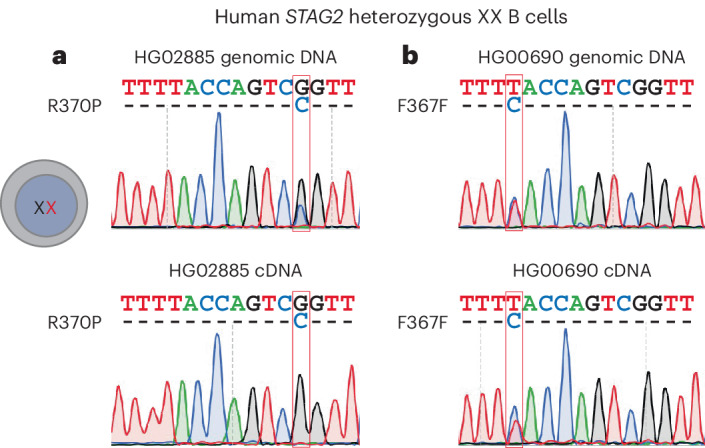
X-linked human genetic variation associated with skewed X chromosome usage in blood-derived polyclonal B lymphoblastoid cells. **a**, Representation of the human *STAG2* R370P missense variant rs777011872 (red rectangle) was determined by Sanger sequencing of gDNA (top) and cDNA (bottom) in a polyclonal B cell line derived from the blood of 1000 Genomes donor HG02885. **b**, Representation of a synonymous human *STAG2* variant, F367F (red rectangle), was determined as in **a** in a polyclonal B cell line derived from the blood of 1000 Genomes donor HG00690.

Mouse models revealed that clones expressing *Stag2* variants failed to contribute to the formation of lymphocytes of XX females. To test the relevance of this finding for human biology, we examined the representation of the human STAG2 rs777011872 R370P variant described in Fig. 1. As expected, both WT and rs777011872 variant sequences were represented in the gDNA of polyclonal B cells derived from the blood of HG02885 (marked by a red rectangle in Fig. 6a, top). By contrast, only WT sequences were detected in cDNA, while the rs777011872 variant was absent (marked by a red rectangle in Fig. 6a, bottom). Consistent with the mouse models, clones expressing variant *STAG2* were therefore underrepresented in human B lymphocytes, indicating that the *STAG2* R370P variant skews the clonal composition of human blood. As a control, we analyzed polyclonal B cells derived from the blood of HG00690 with a synonymous variant (T to C substitution at F367), which does not alter the STAG2 protein sequence. Both the WT and the variant were readily detectable in cDNA (red rectangle in Fig. 6b, bottom) as well as in gDNA (red rectangle in Fig. 6b, top). This indicates that not all sequence variation in *STAG2* necessarily affects the representation of variant-expressing clones in human B lymphocytes.

## Discussion

X-linked genetic variation is ubiquitous in XX individuals and gives rise to intra-individual epigenetic diversity as a result of XCI and its clonal propagation. Here we report how X-linked genetic variation can alter organismal development. *Stag2*^variant^ clones were found enriched in the heart but excluded from the lymphoid compartment. Notably, and in contrast to certain X-linked disease mutations^2,12^, the impact of genetic variation on lymphoid specification and differentiation was due not to an intrinsic inability of *Stag2*^variant^ clones to expand or survive. Instead, it was driven by interactions between WT and variant clones. In the absence of *Stag2*^WT^ cells—namely in hemizygous *Stag2*^variant^ males and homozygous *Stag2*^variant^ females—*Stag2*^variant^ progenitors generated normal numbers of lymphocytes.

Although *Stag2* variants reduce or abolish cohesin–CTCF interactions, *Stag2*^variant^ T and B cells showed WT levels of secondary *Tcra* rearrangements and *Igh* class switch recombination, both of which are cohesin-dependent genomic processes^24–26^. Future work will address whether cohesin–ligand interactions are dispensable for *Tcra* recombination and *Igh* class switch recombination or whether the presence of WT *Stag1* compensates for variant *Stag2* in these processes.

The finding that *Stag2*^WT^ cells exclude *Stag2*^variant^ clones from the lymphoid compartment is reminiscent of classical cell competition paradigms where cells are eliminated not because they have low absolute levels of fitness but rather due to fitness differentials between neighboring cells^27–29^. Current models suggest that cell competition amplifies the impact of small fitness differentials, which can manifest in the expression of ribosomal or mitochondrial genes^27,32^. *Stag2*^variant^ progenitors display deregulated gene expression, including genes related to ribosomal and mitochondrial (dys)function. While genes deregulated in *Stag2*^variant^ cells overlap gene sets implicated in cell competition^27,32^, they are also highly enriched for cohesin binding. To what extent these changes are caused directly by disruption of cohesin–ligand interactions remains to be determined.

The sensing of fitness differentials in cell competition may involve dedicated receptor–ligand systems^29^ or interactions with support systems such as epithelia^29^ or stem cell niches^29,33,34^. The outcome of cell competition is typically that loser cells die by apoptosis and do not contribute to the adult organism^27–29^. By contrast, *STAG2*^variant^ clones contributed to adult cell types and tissues, and their contribution varied from >50% in the heart, ~50% in the brain and <50% in skeletal muscle, to essentially nil in the lymphoid system. Hence, in the scenario examined here, X-linked competition does not eliminate X-linked genetic diversity but determines how this diversity is deployed in organismal development.

Strikingly, *Stag2*^variant^ clones retained their lymphoid potential in the face of competition. Removal of *Stag2* from WT clones at the hematopoietic stem and progenitor cell stage allowed *Stag2*^variant^ clones to progress through lymphoid specification and differentiation and to dominate the lymphoid compartment. Interestingly, in the same individual mice where *Stag2*^variant^ clones dominated the lymphoid compartment*, Stag2*^variant^ clones continued to be outnumbered within the hematopoietic stem and progenitor compartment and hence appeared to be on a loser trajectory. This ‘loser takes all’ behavior was unexpected, as in other forms of cell competition, loser cells are stereotypically eliminated by apoptosis^27–29^. In our experimental setting, therefore, *Stag2*^WT^ cells were continually required to exclude X-linked variants from the lymphoid compartment.

What mechanisms might underlie X-linked competition in hematopoiesis? Stem cells need niches that provide resources such as the stem cell factor (SCF) and the chemokine CXCL12. If such niches are limiting, competition may serve as a mechanism of control^33^. Indeed, leukemic stem cells may outcompete normal HSCs for niche access in the bone marrow^34^. Of note, mRNA for the SCF receptor c-kit and the CXCL12 receptor CXCR4 was reduced in *Stag2*^variant^ progenitors (Supplementary Data 2), which—we speculate—may limit their competitiveness for niche-derived factors in the presence of *Stag2*^WT^. Interestingly, HSCs and lymphoid progenitors may depend on distinct niches^35–37^, which could potentially explain the difference in severity of X-linked competition among stem cells and lymphoid progenitors.

In agreement with our findings in mouse models, *STAG2*^variant^ clones were undetectable in blood-derived human B cells heterozygous for the R370P *STAG2* missense variant rs777011872, suggesting that genetic variation can drive X-linked competition in humans. In support of this conclusion, female patients with mutations in *STAG2* or the X-linked cohesin regulator *HDAC8* typically show heavy skewing of X chromosome usage toward *STAG2*^WT^ clones in blood^38–44^.

In conclusion, noncell-autonomous mechanisms shape the contribution of X-linked clonal diversity across cell types and tissues as the result of clonal interactions. As X-linked genetic variation is common in humans, clonal interactions that shape the deployment of X-linked diversity may be widespread in XX individuals.

## Methods

This study complies with all relevant ethical regulations. The protocols used were approved by the Imperial College London Animal Welfare and Ethical Review Body and were performed according to the Animals (Scientific Procedures) Act under a Project License issued by the UK Home Office.

### Human sequence analysis

We interrogated gnomAD (v2.2.2) for human sequence variation and used dbSNP to identify gnomAD variant X-123185062 as rs777011872. ENSEMBL data slicer (http://www.ensembl.org/Homo_sapiens/Tools/DataSlicer/Edit?db=core;tl=0ZVTRpmGovxkhbJc) was used to query position of X chromosome (chrX): 124051212–124051212 in the 1000 Genomes high coverage variants (http://ftp.1000genomes.ebi.ac.uk/vol1/ftp/data_collections/1000G_2504_high_coverage/working/20201028_3202_raw_GT_with_annot/20201028_CCDG_14151_B01_GRM_WGS_2020-08-05_chrX.recalibrated_variants.vcf.gz). A single instance of rs777011872 was found. Scanning donors with GT of 0/1 or 1/1 (that is, with an alternative allele) identified HG02885 as the donor of this variant. She is part of a trio with daughter HG02886 and husband HG02884, and neither husband nor daughter has the variant. A search of the Coriell repository (https://www.coriell.org/Search?q=HG02885) indicates the availability of DNA and LCLs for HG02885.

### Isothermal calorimetry

STAG2–RAD21 complexes were isolated as described previously^16^. Isothermal calorimetry was performed using a MicroCal iTC 200 (Malvern Panalytical) at 25 °C. STAG21–RAD21 and CTCF peptide ligands were dialyzed overnight at 4 °C against 20 mM Tris (pH 7.7), 150 mM NaCl and 0.5 mM tris(2-carboxyethyl)phosphine. For each titration, 300 μl of 50 μM STAG2–RAD21 was added to the calorimeter cell. CTCF peptide was adjusted to a concentration of 500 μM and injected into the sample cell as 16× 2.5-μl syringe fractions. Results were analyzed and displayed using Origin 7.0 software package supplied with the instrument. Data were analyzed using the one-site binding model.

### Mice

Experiments on mice were performed under a UK Home Office project license and according to the Animals (Scientific Procedures) Act. Mice carrying *Stag2* variants were generated by zygotic co-injections of *Cas9* mRNA (GeneArt, Invitrogen), ssDNA donor template (IDT) and tracrRNA/crRNA (IDT; see Supplementary Table 1 for guide sequences) and maintained on a mixed C57BL/129/CD1 background. The *Atrx*^*Luc*/*βGal*^ reporter allele was generated as described^21,22^. *Stag2*^lox^ (Stag2tm1c(EUCOMM)Wtsi; JAX stock, 030902 (ref. ^30^)) and VavCre (B6.Cg-Tg(VAV1-cre)1Graf/MdfJ; JAX stock, 035670 (ref. ^31^)) and OT-I (C57BL/6-Tg(TcraTcrb)1100Mjb/J; JAX stock, 003831 (ref. ^45^)) mice have been described.

### Antibody staining, flow cytometry analysis and cell sorting

Mouse bone marrow cells were stained for lineage markers using biotinylated CD4, CD8, B220, CD19, NK1.1, CD11b, Ter119 and Gr-1 antibodies, incubated with streptavidin magnetic beads (Miltenyi Biotec, 130-048-102) and depleted using MACS LS columns (Miltenyi Biotec, 130-042-401). To analyze and sort LSKs, c-kit^+^ cells and CLPs, lineage-negative cells were stained with Sca-1-BV510 (BD Biosciences, 565507; 1:50), cKit-PE-Cy7 (Thermo Fisher Scientific, 25-1171-82; 1:100), FLT3-PE (Thermo Fisher Scientific, 12-1351-82; 1:50), CD127 (Thermo Fisher Scientific, 17-1271-82; 1:50) and streptavidin-eFluor 450 (eBioscience, 48-4317-82; 1:100). To isolate B cell progenitors, bone marrow cells were depleted of Ter119, CD11b and Gr-1 and stained with B220-FITC (BD Biosciences, 553088; 1:100), PE antimouse CD19 (BD Biosciences, 557399; 1:100), IgM-BV421 (BioLegend, 406517; 1:100) and CD43-APC (BD Biosciences, 560663; 1:100) antibodies. Mature monocytes and granulocytes were sorted from total bone marrow stained with CD11b-APC (BioLegend, 101212; 1:100) and Ly6-G-FITC (BD Biosciences, 561105; 1:100) antibodies. Thymocytes were stained with anti-CD4-BV421 (BioLegend, 100438; 1:300), CD8-APC (BioLegend, 17-0081-83; 1:300), CD25-PE (BioLegend, 102007; 1:100) and TRCβ-FITC (BD Biosciences, 553171; 1:100). Lymph node cells were stained with B220-BV421 (BioLegend, 103240; 1:100) and CD4-PE (BioLegend, 100512; 1:300) or CD4-APC (Thermo Fisher Scientific, 17-0041-83; 1:300). Cell populations were analyzed using a Fortessa Flow Cytometer (BD Biosciences) and sorted using a BD Aria Fusion or Aria III (see Supplementary Table 2 for details).

### Live-cell reporter assays

Thymocytes, lymphocytes and bone marrow cells were isolated, and bone marrow was depleted of cells expressing the lineage markers CD4, CD8, B220, CD19, NK1.1, CD11b, Ter119 and GR-1. To detect βGal activity, 1 mM of nonfluorescent FDG (Thermo Fisher Scientific, F1179) substrate was delivered into the cells by hypotonic loading at 37 °C. In total, 2 × 10^6^–2 × 10^7^ cells in 100 μl PBS, 2% FBS and 10 mM HEPES (Merck, H0887) were prewarmed to 37 °C, and 100 μl of prewarned FDG solution was added to 100 μl of cells for 1 min. To stop FDG loading, samples were placed on ice, and 2 ml of ice-cold PBS, 2% FBS and 10 mM HEPES were added. Following 45-min incubation on ice, cells were stained for surface markers as described above, and the conversion of FDG into FITC was detected by flow cytometry. All experiments included cells lacking the *Atrx*^*Luc*/*βGal*^ reporter as negative controls.

### Cell line culture and genetic engineering of HAP1 cells

Epstein-Barr Virus-transformed B lymphoblastoid cells (Coriell Institute for Medical Research) were maintained in Roswell Park Memorial Institute-1640 (RPMI-1640) medium supplemented with 15% foetal calf serum (FCS), 2 mM l-glutamine and 1% penicillin–streptomycin (Pen–Strep). HAP1 cells^46^ were cultured in Iscove’s Modified Dulbecco’s Medium (IMDM, Invitrogen) supplemented with 10% FCS (Clontech), 1% Pen–Strep (Invitrogen) and 1% UltraGlutamin (Lonza). Mutant cells were generated by CRISPR–Cas9 technology. Guide RNAs were annealed into pX330. To mutate the locus of interest, we cotransfected the repair oligonucleotide with the desired mutation as well as a silent mutation (see Supplementary Table 1 for primer sequences).

### T cell culture and cell proliferation assay

Round-bottom 96-well plates were coated overnight at 4 °C with purified anti-TCRβ chain clone H57 (BD Biosciences, 553167) in PBS with MgCl_2_ and CaCl_2_ (Sigma, D8662-1L). Thymocyte cell suspensions were incubated in the plates for 16–18 h in IMDM media (Gibco, 12440-053) with 10% FBS, 1% l-glutamine, 1% Pen–Strep, 1% sodium pyruvate, 0.1% 2-mercaptoethanol and 2 µg ml^−1^ soluble anti-CD28 (BioLegend, 102102). Thymocyte cell proliferation was tracked using CellTrace CFSE Cell Proliferation Kit (Thermo Fisher Scientific, C34554) according to the manufacturer’s instructions after 3 days of incubation. Cells were stained with PE anti-CD4 (BioLegend, 100512; 1:300), APC anti-CD8a (Thermo Fisher Scientific, 17-0081-83; 1:300) and BV421 anti-CD69 (BD Biosciences, 562920; 1:50). Single cells were sorted using the FACSAria Fusion Flow Cytometer (BD Biosciences). Flow cytometry FCS files were analyzed with FlowJo v10 (TreeStar).

### RNA extraction, reverse transcription and allele-specific qPCR

RNA was extracted from sorted cells using the RNeasy Plus Micro Kit (Qiagen) according to the manufacturer’s instructions. Tissue samples were lysed in Trizol and homogenized using a TissueLyser II (Qiagen) and 5 mm stainless steel beads (Qiagen) for 4 min at 24,000 rpm. Tissue homogenates were extracted with chloroform. RNA isolation from tissue homogenates was performed using the RNeasy Mini Kit (Qiagen), according to the manufacturer’s instructions. cDNA was synthesized using SuperScript III reverse transcriptase (Thermo Fisher Scientific) following the manufacturer’s instructions, with 10 µM random primers. Allele-specific qPCR assays were performed with TaqMan Fast Universal Master Mix (Thermo Fisher Scientific) and run on a CFX96 real-time PCR machine (Bio-Rad). Allele-specific primers and fluorescent TaqMan probes were used to discriminate between WT and variant alleles. Real-time PCR data were collected and analyzed using CFX Maestro 1.1 Software (Bio-Rad). Percentages of WT and variant mRNA were calculated based on the normalized Δ*C*_*T*_ values between amplification with WT and variant TaqMan probes (see Supplementary Table 1 for primer sequences and TaqMan probes).

### Sanger sequencing

gDNA was isolated from sorted cells and tissue samples using the DNeasy Blood and Tissue Kit (Qiagen), following the manufacturer’s instructions. gDNA and cDNA were amplified by PCR followed by Sanger sequencing (see Supplementary Table 1 for primer sequences).

### scRNA-seq

Bone marrow cells were depleted of lineage-positive cells, loaded with FDG as described above, and then stained with antibodies against Sca-1-BV510, cKit-PE-Cy7, FLT3-PE CD127-APC and streptavidin-eFluor 450. FITC^+^ and FITC^−^ progenitor cells were sorted and loaded on the 10X Genomics Chromium System. scRNA-seq libraries were prepared using Chromium Single Cell 3′ Reagent Kits User Guide (v2 Chemistry), sequenced on a NextSeq 2000 (100 cycles; Illumina), and 10X Genomics CellRanger (v5.0.1) was used for barcode splitting, UMI (unique molecular identifier) counting and alignment to the mouse genome (GRCm38, Ensembl 107 annotations). Quality control and subsequent analysis were conducted in R using Seurat (v4.3.0.1)^47^. Cells with aberrant feature counts or mitochondrial sequence fractions were discarded using data-driven filter criteria (two median absolute deviations on either side of the median values). For each sample individually, the structure was assessed using a subset of 2,000 variable genes (identified using the FindVariableFeatures function) that were used to identify the principal component analysis (PCA) dimensionality for downstream Uniform Manifold Approximation and Projection (UMAP) analysis^48^. Samples were integrated using genes identified by the Seurat FindIntegrationAnchors function. Progenitors were identified using gene lists from scType^49^ supplemented with markers for bone marrow progenitors (Supplementary Data 1). Differential expression analysis was conducted using DESeq2 (v1.42.0) with a threshold of adjusted *P* < 0.01. Gene ontology analyses were conducted using clusterProfiler (v4.10.0)^50^ with a threshold of adjusted *P* < 0.05. Annotation of the lineage-primed clusters was performed using AUCell^51^ combined with manual annotation using marker genes provided in Supplementary Data 4. The observed versus expected numbers of WT and variant cells in each cluster were tested by bootstrapped permutation tests (1,000 iterations) using scProportionTest in R^52^. Classification of cell cycle stages was implemented in R using Seurat (v4.1.0)^47^.

### ChIP–seq and analysis of cohesin binding

*STAG1* and *STAG2* gene editing and chromatin immunoprecipitation using mouse anti-RAD21 (Millipore, 05-908; 10 μg per ChIP) were done as was described^16^. DNA was sheared using Biorupter Pico (Diagenode), five cycles of 15-s on and 90-s off. Reads were trimmed using TrimGalore (v.0.6.0)^53^, mapped to hg19 using Bowtie 2 (v.2.3.4)^54^ with default settings. Bigwig files were generated with DeepTools (v.3.1.3)^55^ with the following settings: minimum mapping quality of 15, bin length of 10 bp, extending reads to 200 bp and reads per kilobase per million reads normalization. Heatmaps were generated using DeepTools on previously called RAD21 peaks^16^. Reads for cohesin SMC1 ChIP–seq from hematopoietic progenitors^56^ (GSM3790131) were trimmed with cutadapt (10.14806/ej.17.1.200) and aligned to mm10 with Bowtie 2 (ref. ^54^). Duplicates were removed with Picard 2.27.5 (https://broadinstitute.github.io/picard/) and peaks called with MACS3 3.0.0b1 (ref. ^57^). Promoters with SMC1 peaks <2 kb from the transcription start site were called cohesin-associated. Heatmaps were produced using the genomation toolkit^58^. Odds ratios and *P* values were calculated using Fisher’s exact test.

### Analysis of *Tcra* locus rearrangement and serum immunoglobulin isotypes

gDNA from sorted double-positive (DP) thymocytes was isolated using DNeasy Blood and Tissue Kits (Qiagen). Threefold serial dilutions of gDNA were amplified using a forward Vα8 primer and reverse primers for Jα61 or Jα22 as described previously^24^. *Cd14* was the genomic control (see Supplementary Table 1 for primer sequences). Concentrations of serum immunoglobulin isotypes in adult unimmunized mice were determined by enzyme-linked immunosorbent assay as advised by the manufacturers (Thermo Fisher Scientific; IgM: 88-50470-22, IgG2a: 88-50420-22, IgG2b: 88-50430-22 and IgG3: 88-50440-22).

### Statistics and reproducibility

No statistical method was used to predetermine the sample size. No data were excluded from the analyses. The experiments were not randomized, as sample allocation into different groups was defined by genotype. The investigators were not blinded to allocation during experiments and outcome assessment. ChIP–seq peaks were called in MACS3, and odds ratios and *P* values were calculated by Fisher’s exact test. Flow cytometry statistics were done in FlowJo. Statistical analysis of differential gene expression in scRNA-seq experiments was performed by DESeq2. Statistical analysis of cell frequencies was done by bootstrapped permutation tests using scProportionTest in R^52^. Statistical analysis of allelic representation was done in Prism.

### Reporting summary

Further information on research design is available in the Nature Portfolio Reporting Summary linked to this article.

## Online content

Any methods, additional references, Nature Portfolio reporting summaries, source data, extended data, supplementary information, acknowledgements, peer review information; details of author contributions and competing interests; and statements of data and code availability are available at 10.1038/s41588-024-01840-5.

### Supplementary information

Supplementary Information Supplementary Tables 1 and 2.

Reporting Summary

Supplementary Data 1 Marker genes for the annotation of multipotent and lineage-restricted progenitors.

Supplementary Data 2 Differentially expressed genes in multipotent and lineage-restricted progenitors.

Supplementary Data 3 Gene ontology terms enriched among upregulated and downregulated genes in merged progenitors.

Supplementary Data 4 Marker genes for the annotation of lineage-primed progenitors.

### Source data

Source Data Fig. 1 Numerical source data.

Source Data Fig. 2 Statistical source data.

Source Data Fig. 3 Statistical source data.

Source Data Fig. 4 Statistical source data.

Source Data Fig. 5 Statistical source data.

Source Data Extended Data Fig. 4 Statistical source data.

Source Data Extended Data Fig. 6 Statistical source data.

Source Data Extended Data Fig. 7 Statistical source data.

Source Data Extended Data Fig. 9a Unprocessed and processed gel.

Source Data Extended Data Fig. 9b,c Statistical source data.

## Data Availability

High-throughput sequencing data generated in this study are available from the NCBI Gene Expression Omnibus (GEO) under accession GSE261622. Source data are provided with this paper.

## Code availability

No custom code was generated for this study.
