# Supplementary material for: Competition shapes the landscape of X-chromosome-linked genetic diversity
Source: Nat Genet. 2024 Jul 26;56(8):1678–88. doi: 10.1038/s41588-024-01840-5 (PMC11319201; doi:10.1038/s41588-024-01840-5)
Supplement: Supplementary file 1 — Supplementary Tables 1 and 2. [file 41588_2024_1840_MOESM1_ESM.pdf]

nature genetics

Supplementary information

https://doi.org/10.1038/s41588-024-01840-5

Competition shapes the landscape of X-chromosome-linked genetic diversity

In the format provided by the authors and unedited

Supplementary Table 1

Coding variation on the human X chromosome versus human autosomes

	Gene #	Obs. variants*	Expected variants**	Observed/ expected
X Chr	n=727			
Synonymous		39453	39021	1.011
Missense		70048	88265	0.794
Loss of Function		1652	8518	0.194
Autosomes	n=17484			
Synonymous		1528646	1549596	0.987
Missense		3032372	3406415	0.890
Loss of Function		127689	319504	0.400

* Number of rare variants observed in ExAC r0.3, allele frequency < 0.1% (ref 8).
** Depth-adjusted number of expected rare variants, allele frequency < 0.1% (ref 8).

Oligonucleotide sequences

Guide RNAs

W334: 5'- GCTATTTAAAATATGTTGGT
R370: 5'- AAAACTAACCTTGAATCGAC
Primer sequences:
Stag2 R370Q Fwd 5'-ATGTCTTACTGCTTTACAAGGGC-3'
Stag2 R370Q Rev 5'- TGCAGTTAGAACTTCTTCACTACTC-3'
Stag2 W334A Fwd 5'- GGGATGCAATAGCTGAAATCAGAG-3'
Stag2 W334A Rev 5'-AGCCCTTGTAAAGCAGTAAGACA-3'

TaqMan probes:

R370Q WT (HEX-TCTTTACCAGTCGATTCAAGGATA-BHQ)
R370Q variant (6-FAM-TCTTTACCAGTCAATTCAAGGATAG-BHQ)
W334A WT (HEX-AATATGTTGGTTGGACTATGCATGA-BHQ)
W334A variant (6-FAM-ATATGTTGGTGCGACTATGCATG-BHQ).

Genomic DNA was amplified by PCR with the following allele-specific primers:

Stag2 W334A Fwd 5'-ATGCCCAGTCGTACAGAAGC-3'
Rev 5'-TGGCCAAAAGTACACATCCA-3'
Stag2 R370Q Fwd 5'-TCTTACTGCTTTACAAGGGCTTT-3'
Rev 5'-AGATGGGACAAATGGATGGA-3'.

cDNA was PCR-amplified with the following allele-specific primers:

Stag2 W334A Fwd 5'- GCTAACGAAAGGCTAGAACTCC-3'
Rev 5'-ACATTTTCACAATCTTCTGCAGT-3'
Stag2 R370Q Fwd 5'-GCATTGAAGAGATTGGCATTTGG-3'
Rev 5'-AGAAATTCTCCAGCTGCAACT-3'

gDNA primers used to analyse *Tcra* locus rearrangements

Vα8 Fwd: 5´-CAGACAGAAGGCCTGGTCAC- 3'
Jα61 Rev: 5´-ATGAGTCTTCCAGTCATGGC-3´
Jα22 Rev: 5´-TGTCAGTTGGGTTCCAGATCC-3´
Cd14 Fwd: 5´-GCTCAAACTTTCAGAATCTACCGAC-3´
Rev: 5´-AGTCAGTTCGTGGAGGCCGGAAATC-3´

Targeting *STAG1* and *STAG2* in HAP1 cells

STAG1 guide RNA 5′- TTGGCTGGACTCTTCATGAC-3′
STAG2 guide RNA 5′-GACAGTTATTTAAAATATGT-3′
STAG1 W337A repair oligo 5′-
AGTACTGAGACAAACATAACTTCCATCAAAGCTTAGAACAGAGTAACTTACCCTGTCGTGAAGAGTAG
CGCCAACATATTTTAGGTAACTGTCATTTAGGAAGGCATCACTATACATTTTCATC-3′
STAG2 W334A repair oligo 5′-
CTTAATGACAGTTATTTAAAATATGTTGGTGCGACTATGCATGATAAGGTAAGATGTGCCCTTCAGAC
TGCTTCTTTCTATACATCGGCGTGGCTGTCTGCACCTCTCATTCATGAG-3′

Supplementary Table 2 - Details of Antibodies, Reagents, Software and Databases used

Antibodies		
Hamster anti-mouse TCR β Chain	BD Biosciences	Cat#553167; RRID:AB_394679
Hamster purified anti-mouse CD28	BioLegend	Cat#102102; RRID:AB_312867
Hamster Anti-Mouse CD69	BD Biosciences	Cat# 562920, RRID:AB_2687478
Rat anti-mouse CD4	BioLegend	Cat# 100512, RRID:AB_312715
Rat anti-mouse CD8a	Thermo Fisher Scientific	Cat# 17-0081-83, RRID:AB_469336
Mouse anti-RAD21	Millipore	Cat# 05-908
Hamster anti-mouse TCR β Chain	BD Biosciences	Cat#553167; RRID:AB_394679
BV510 anti-mouse Ly-6A/E/Sca-1	BD Biosciences	Cat# 565507
PE-Cy7 anti-human/mouse CD117/cKit	Thermo Fisher Scientific	Cat# 25-1171-82
PE anti-mouse CD135/FLT3	Thermo Fisher Scientific	Cat# 12-1351-82
APC anti-mouse CD127/IL-7Rα	eBioscience	Cat# 17-1271-82
eFluor 450 anti-mouse streptavidin	eBioscience	Cat# 48-4317-82
FITC anti-mouse CD45R/B220	BD Biosciences	Cat# 553088
BV421 anti-mouse/human CD45R/B220	Biolegend	Cat# 103240
BV421 anti-mouse IgM	Biolegend	Cat# 406517
APC anti-mouse CD43	BD Biosciences	Cat# 560663
APC anti-human/mouse Cd11b	Biolegend	Cat# 101212
FITC anti-mouse Ly-6G	BD Biosciences	Cat# 561105
BV421 anti-mouse CD4	Biolegend	Cat# 100438
PE anti-mouse CD4	Biolegend	Cat# 100512
APC anti-mouse CD8a	Biolegend	Cat# 17-0081-83
PE anti-mouse CD25	Biolegend	Cat# 102007
FITC anti-mouse TRCβ	BD Biosciences	Cat# 553171

Chemicals, peptides, and recombinant proteins		
16% Formaldehyde (w/v), Methanol-free	Pierce	Cat# 28908
FDG	Thermo Fisher Scientific	Cat# F1179
Taqman^{TM} Universal PCR Master Mix	Thermo Fisher Scientific	Cat# 4324018
Streptavidin MicroBeads	Miltenyi Biotec	Cat# 130-048-101
LS columns	MiltenyiBiotec	Cat# 130-042-401
16% Formaldehyde (w/v), Methanol-free	Pierce	Cat# 28908
CellTrace™ CFSE Cell Proliferation Kit	ThermoFisher Scientific	Cat#C34554

Deposited data		
scRNA-seq data	This Study	GSE240997, reviewer token wpcfeiygthqzfej
SMC1 ChIP-seq	Ochi et al., 2020	GSM3790131
RAD21 ChIP-seq	This study	GSE261621, reviewer token wpcfeiygthqzfej

Experimental models: Organisms/strains		
Mouse: Stag2 lox	JAX stock #030902	https://www.jax.org/strain/030902
Mouse: VavCre	JAX stock #035670	https://www.jax.org/strain/035670
Mouse: OT-I	JAX stock #003831	https://www.jax.org/strain/003831
Human cell line HAP1	Carette at al., 2011	

Software and algorithms		
scType	Stuart et al., 2019	https://sctype.app/
R	The R Foundation	https://www.r-project.org/
Seurat v4.1.0	Hao et al., 2021	https://satijalab.org/seurat/
clusterProfiler	Wu et al., 2021	https://bioconductor.org/packages/release/bioc/html/clusterProfiler.html
Bowtie 2	Langmead and Salzberg, 2012	
Cutadapt		DOI:10.14806/ej.17.1.200
Picard		https://broadinstitute.github.io/picard/
Genomation	Akalin et al., 2014	

Databases		
gnomAD	Broad Institute	https://gnomad.broadinstitute.org/
dbSNP	NCBI	https://www.ncbi.nlm.nih.gov/snp/
dbSNP	NCBI	https://www.ncbi.nlm.nih.gov/snp/
Immgen	Immunological Genome Project	www.immgen.org
Haemosphere		https://www.haemosphere.org
