# Supplementary material for: Competition shapes the landscape of X-chromosome-linked genetic diversity
Source: Nat Genet. 2024 Jul 26;56(8):1678–88. doi: 10.1038/s41588-024-01840-5 (PMC11319201; doi:10.1038/s41588-024-01840-5)
Supplement: Supplementary file 2 — Reporting Summary [file 41588_2024_1840_MOESM2_ESM.pdf]

nature portfolio

Corresponding author(s): M Merkenschlager

Last updated by author(s): May 13, 2024

Reporting Summary

Nature Portfolio wishes to improve the reproducibility of the work that we publish. This form provides structure for consistency and transparency in reporting. For further information on Nature Portfolio policies, see our Editorial Policies and the Editorial Policy Checklist.

Statistics

For all statistical analyses, confirm that the following items are present in the figure legend, table legend, main text, or Methods section.

n/a	Confirmed	
☐	☒	The exact sample size (*n*) for each experimental group/condition, given as a discrete number and unit of measurement
☐	☒	A statement on whether measurements were taken from distinct samples or whether the same sample was measured repeatedly
☐	☒	The statistical test(s) used AND whether they are one- or two-sided<br>*Only common tests should be described solely by name; describe more complex techniques in the Methods section.*
☒	☐	A description of all covariates tested
☐	☒	A description of any assumptions or corrections, such as tests of normality and adjustment for multiple comparisons
☐	☒	A full description of the statistical parameters including central tendency (e.g. means) or other basic estimates (e.g. regression coefficient) AND variation (e.g. standard deviation) or associated estimates of uncertainty (e.g. confidence intervals)
☐	☒	For null hypothesis testing, the test statistic (e.g. *F*, *t*, *r*) with confidence intervals, effect sizes, degrees of freedom and *P* value noted<br>*Give P values as exact values whenever suitable.*
☒	☐	For Bayesian analysis, information on the choice of priors and Markov chain Monte Carlo settings
☒	☐	For hierarchical and complex designs, identification of the appropriate level for tests and full reporting of outcomes
☒	☐	Estimates of effect sizes (e.g. Cohen's *d*, Pearson's *r*), indicating how they were calculated

Our web collection on statistics for biologists contains articles on many of the points above.

Software and code

Policy information about availability of computer code

Data collection	Real time PCR data was collected and analysed using Bio-Rad CFX Maestro 1.1 Software.<br>scRNA seq libraries were prepared using Chromium Single Cell 3' Reagent Kits User Guide v2 Chemistry, sequenced on an Illumina NextSeq 2000 (100cycles) and 10x Genomics CellRanger v5.0.1 was used for barcode splitting,UMI counting, and alignment to the mouse genome (GRCm38, Ensembl 107 annotations).Chip-seq Reads were trimmed using TrimGalore v.0.6.0, mapped to hg19 using Bowtie2 v.2.3.4. Bigwig files were generated with DeepTools v.3.1.3. Reads for cohesin SMC1 ChIP-seq from haematopoietic progenitors were trimmed with cutadapt and aligned to mm10 with Bowtie 2. Duplicates were removed with Picard (2.27.5) and peaks called with MACS3. Heatmaps were produced using the genomation toolkit or DeepTools. BD FACSDiva Sotfware was used to collect flow cytometry data.
Data analysis	Single cell RNA-sequencing analysis and quality control was conducted in R using Seurat v4.3.0.1. FindVariableFeatures function was used to identify the most variable genes. Samples were integrated using genes identified by the Seurat FindIntegrationAnchors function. Progenitors were identified using gene lists from scType supplemented with markers for bone marrow progenitors (Supplementary_Data_1). Annotation of the lineage-primed clusters was performed using AUCell v1.24.0 combined with manual annotation using marker genes provided in Supplementary_Data_4.Classification of cell cycle stages was implemented in R using Seurat v4.1.0. Differential expression analysis was performed using DESeq2 v1.42.0. Gene Ontology analyses were conducted using clusterProfile v4.10.0 (Wu et al., 2021). FDR for figures 3c,d and e were determined by permutation test using scProportionTest in R. Flow cytometry data was analysed using Flowjo v.1O. Statistical analysis in Figure 2 and 4 was performed using Graphpad Prism v.9.

For manuscripts utilizing custom algorithms or software that are central to the research but not yet described in published literature, software must be made available to editors and reviewers. We strongly encourage code deposition in a community repository (e.g. GitHub). See the Nature Portfolio guidelines for submitting code & software for further information.

Data

Policy information about availability of data

All manuscripts must include a data availability statement. This statement should provide the following information, where applicable:

- Accession codes, unique identifiers, or web links for publicly available datasets
- A description of any restrictions on data availability
- For clinical datasets or third party data, please ensure that the statement adheres to our policy

High throughput sequencing data generated in this study are available from the NCBI Gene Expression Omnibus (GEO) under accession number GSE261622 (https://www.ncbi.nlm.nih.gov/geo/query/acc.cgi?acc=GSE261622).
The scRNA-seq data generated in this study have been deposited in the NCBI Gene Expression Omnibus (GEO) under accession number GSE240997 (https://www.ncbi.nlm.nih.gov/geo/query/acc.cgi?acc=GSE240997).
The Chip-seq data generated in this study have been deposited in the NCBI Gene Expression Omnibus (GEO) under accession number GSE261621 (https://www.ncbi.nlm.nih.gov/geo/query/acc.cgi?acc=GSE261621).
SMC1 Chip-seq data used in this study (Ochi et al., 2020): GSM3790131 (https://www.ncbi.nlm.nih.gov/geo/query/acc.cgi?acc=GSE131583).
The following databases where used in this study: gnomAD (https://gnomad.broadinstitute.org/), dbSNP (https://www.ncbi.nlm.nih.gov/snp/), Metadome (https://stuart.radboudumc.nl/metadome/dashboard), GRCm38/mm10 (https://www.ncbi.nlm.nih.gov/datasets/genome/GCF_000001635.20/), Haemosphere (https://www.haemosphere.org.) and Immgen (www.immgen.org).

Research involving human participants, their data, or biological material

Policy information about studies with human participants or human data. See also policy information about sex, gender (identity/presentation), and sexual orientation and race, ethnicity and racism.

Reporting on sex and gender	N/A
Reporting on race, ethnicity, or other socially relevant groupings	N/A
Population characteristics	N/A
Recruitment	N/A
Ethics oversight	N/A

Note that full information on the approval of the study protocol must also be provided in the manuscript.

Field-specific reporting

Please select the one below that is the best fit for your research. If you are not sure, read the appropriate sections before making your selection.

☒ Life sciences ☐ Behavioural & social sciences ☐ Ecological, evolutionary & environmental sciences

For a reference copy of the document with all sections, see nature.com/documents/nr-reporting-summary-flat.pdf

Life sciences study design

All studies must disclose on these points even when the disclosure is negative.

Sample size	No statistical test was used to determine sample size. Sample sizes were chosen based on common standards of the field.
Data exclusions	Single cell RNA sequencing: Cells with aberrant feature counts or mitochondrial sequence fraction were discarded using data-driven filter criteria (2 median absolute deviations either side of the median values).
Replication	The number of biological replicates is detailed in the figure legend. Biological replicates are displayed as individual data points in the figures.
Randomization	No randomization was required. Control and variant mice were matched by age and sex. Similar numbers of control and variant mice were included in each experimental session.
Blinding	No blinding was done since the study did not involve any treatment. Experiments done with heterozygous XX individuals did not require blinding since the experiment involved internal controls.

Reporting for specific materials, systems and methods

We require information from authors about some types of materials, experimental systems and methods used in many studies. Here, indicate whether each material, system or method listed is relevant to your study. If you are not sure if a list item applies to your research, read the appropriate section before selecting a response.

Materials & experimental systems

n/a	Involved in the study
☐	☒ Antibodies
☐	☒ Eukaryotic cell lines
☒	☐ Palaeontology and archaeology
☐	☒ Animals and other organisms
☒	☐ Clinical data
☒	☐ Dual use research of concern
☒	☐ Plants

Methods

n/a	Involved in the study
☐	☒ ChIP-seq
☐	☒ Flow cytometry
☒	☐ MRI-based neuroimaging

Antibodies

Antibodies used

Flow cytometry antibodies: Hamster anti-mouse CD28 (BioLegend, Cat#102102, clone:37.51, 2ug/ml), Hamster Anti-Mouse CD69 (BD Biosciences, Cat# 562920, clone:H12F3, 1:50), Rat anti-mouse CD4 (BioLegend, Cat# 100512, clone:RM4-5, 1:300), Rat anti-mouse CD8a (Thermo Fisher Scientific ,Cat# 17-0081-83, clone:53-6.7, 1:300), BV510 anti-mouse Ly-6A/E/Sca-1 (BD Biosciences, Cat# 565507, clone :D7, 1:50), PE-Cy7 anti-human/mouse CD117/cKit (Thermo Fisher Scientific,Cat# 25-1171-82, clone:2B8, 1:100), PE anti-mouse CD135/FLT3 (Thermo Fisher Scientific, Cat# 12-1351-82, clone:A2F10, 1:50), APC anti-mouse CD127/IL-7Rα (eBioscience, Cat# 17-1271-82, clone: A7R34, 1:50), eFluor 450 anti-mouse streptavidin (eBioscience, Cat# 48-4317-82, 1:100), FITC anti-mouse CD45R/B220 (BD Biosciences, Cat# 553088, clone:RA3-6B2, 1:100) , PE anti-mouse CD19 (BD Bioisceinces, Cat# 557399, clone: 1D3, 1:100), BV421 anti-mouse/human CD45R/B220 (Biolegend, Cat# 103240, clone: RA3-6B2, 1:100), BV421 anti-mouse IgM (Biolegend, Cat# 406517,clone:RMM-1, 1:100), APC anti-mouse CD43 (BD Biosciences, Cat# 560663, clone: S7, 1:100), APC anti-human/mouse Cd11b (Biolegend, Cat# 101212 clone: M1/70, 1:100), FITC anti-mouse Ly-6G (BD Biosciences, Cat# 561105, clone: 1A8, 1:100), BV421 anti-mouse CD4 (Biolegend, Cat# 100438, clone: GK1.5, 1:100), PE anti-mouse CD4 (Biolegend, Cat# 100512, clone: RM4-5, 1:300), PE anti-mouse CD25 (Biolegend, Cat# 102007, clone: PC61, 1:100) FITC anti-mouse TRCβ (BD Biosciences, Cat# 553171, clone: H57-597, 1:100), APC anti-mouse CD8a (Biolegend, Cat# 17-0081-83, clone: 53-6.7 , 1:300), APC anti-mouse CD4 (Thermo Fisher, Cat# 17-0041-83, clone : GK1.5, 1:300);
ChIP-seq antibodies: anti-RAD21 (Millipore, Cat# 05-908, 10 ug per ChIP).

Validation

Antibodies used for flow cytometry were purchased from commercial suppliers and were validated by the supplier. Details of the antibodies are provided in the Methods. Validation information can be found at the following links:
Hamster anti-mouse TCR β Chain: https://www.bdbiosciences.com/en-gb/products/reagents/flow-cytometry-reagents/research-reagents/single-color-antibodies-ruo/purified-hamster-anti-mouse-tcr-chain.553167
Hamster anti-mouse CD28: https://www.biolegend.com/en-gb/products/purified-anti-mouse-cd28-antibody-117
Hamster Anti-Mouse CD69: https://www.bdbiosciences.com/en-gb/products/reagents/flow-cytometry-reagents/research-reagents/single-color-antibodies-ruo/bv421-hamster-anti-mouse-cd69.562920
Rat anti-mouse CD4: https://www.biolegend.com/en-gb/products/pe-anti-mouse-cd4-antibody-482
Rat anti-mouse CD8a: https://www.thermofisher.com/antibody/product/CD8a-Antibody-clone-53-6-7-Monoclonal/17-0081-82
BV510 anti-mouse Ly-6A/E/Sca-1: https://www.bdbiosciences.com/en-gb/products/reagents/flow-cytometry-reagents/research-reagents/single-color-antibodies-ruo/bv510-rat-anti-mouse-ly-6a-e.565507
PE-Cy7 anti-human/mouse CD117/cKit : https://www.thermofisher.com/antibody/product/CD117-c-Kit-Antibody-clone-2B8-Monoclonal/25-1171-82
PE anti-mouse CD135/FLT3 : https://www.thermofisher.com/antibody/product/CD135-Flt3-Antibody-clone-A2F10-Monoclonal/12-1351-82
APC anti-mouse CD127/IL-7Rα : https://www.thermofisher.com/antibody/product/CD127-Antibody-clone-A7R34-Monoclonal/17-1271-82
eFluor 450 anti-mouse streptavidin : https://www.thermofisher.com/order/catalog/product/48-4317-82?gclid=CjwKCAjw88yxBhBWEiwA7cm6pVxDc_TunZvrVmeLD_BHNjrM4sKHtS-eVAhNznqKPyfgtSSf8ArRGBoCxlgQAvD_BwE&ef_id=CjwKCAjw88yxBhBWEiwA7cm6pVxDc_TunZvrVmeLD_BHNjrM4sKHtS-eVAhNznqKPyfgtSSf8ArRGBoCxlgQAvD_BwE:G:s&s_kwcid=AL!3652!3!278870232429!!!g!!!1454324556!63404918784&cid=bid_pca_frg_r01_co_cp1359_pjt0000_bid00000_0se_gaw_dy_pur_con&gad_source=1
FITC anti-mouse CD45R/B220: https://www.bdbiosciences.com/en-eu/products/reagents/flow-cytometry-reagents/research-reagents/single-color-antibodies-ruo/fitc-rat-anti-mouse-cd45r-b220.553088
PE anti-mouse CD19 :https://www.bdbiosciences.com/en-gb/products/reagents/flow-cytometry-reagents/research-reagents/single-color-antibodies-ruo/pe-rat-anti-mouse-cd19.557399
BV421 anti-mouse/human CD45R/B220: https://www.biolegend.com/en-gb/products/brilliant-violet-421-anti-mouse-human-cd45r-b220-antibody-7158
BV421 anti-mouse IgM: https://www.biolegend.com/en-gb/products/brilliant-violet-421-anti-mouse-igm-7254
APC anti-mouse CD43: https://www.bdbiosciences.com/en-gb/products/reagents/flow-cytometry-reagents/research-reagents/single-color-antibodies-ruo/apc-rat-anti-mouse-cd43.560663
APC anti-human/mouse Cd11b : https://www.biolegend.com/en-gb/products/apc-anti-mouse-human-cd11b-antibody-345?GroupID=BLG10530
FITC anti-mouse Ly-6G : https://www.bdbiosciences.com/en-eu/products/reagents/flow-cytometry-reagents/research-reagents/single-color-antibodies-ruo/fitc-rat-anti-mouse-ly-6g.561105
BV421 anti-mouse CD4 :https://www.biolegend.com/en-gb/products/brilliant-violet-421-anti-mouse-cd4-antibody-7142?GroupID=BLG4745
PE anti-mouse CD4: https://www.biolegend.com/en-gb/products/pe-anti-mouse-cd4-antibody-482

PE anti-mouse CD25: https://www.biolegend.com/en-gb/sean-tuckers-tests/pe-anti-mouse-cd25-antibody-424?GroupID=BLG10428
FITC anti-mouse TRCβ: https://www.bdbiosciences.com/en-us/products/reagents/flow-cytometry-reagents/research-reagents/single-color-antibodies-ruo/fitc-hamster-anti-mouse-tcr-chain.553171
APC anti-mouse CD8a: https://www.biolegend.com/en-gb/products/apc-anti-mouse-cd8a-antibody-150?GroupID=BLG6765
APC anti-mouse CD4: https://www.thermofisher.com/antibody/product/CD4-Antibody-clone-GK1-5-Monoclonal/17-0041-82
Chip-seq: validation statement for anti-RAD21 antibody can be found in the following link: https://www.merckmillipore.com/GB/en/product/Anti-RAD21-Antibody,MM_NF-05-908

Eukaryotic cell lines

Policy information about cell lines and Sex and Gender in Research

Cell line source(s)	EBV-transformed B lymphoblastoid human cell lines were obtained from Coriell institute for medical research. EBV-transformed B lymphoblastoid human cell lines were derived from XX individuals.<br>HAP1 cells from Carette et al., Nature 2011, a gift from the authors.<br>HAP1 STAG1W337A and STAG2W334A cells were generated in Benjamin Rowland's lab (see paper García-Nieto A, et al. S, Nat Struct Mol Biol, 2023)
Authentication	Genomic DNA was extracted from B lymohoblastoid cell lines and sequenced by Sanger sequencing to verify that they belong to the correct donors . For donor HG02885 STAG2 R370P missense variant rs777011872 was identified and for donor HG00690 synonymous STAG2 variant F367F was identified. HAP1 cells were identified by Karyotyping. Mutants were confirmed by Sanger sequencing
Mycoplasma contamination	Cell lines were purchased from Coriell institute and they are free of mycoplasma contamination (https://www.coriell.org/0/Sections/Support/Global/Lymphoblastoid.aspx?PgId=213). Mycoplasma testing was not performed in the lab as genomic DNA and RNA samples were collected 48 h after arrival of the cell lines. HAP1 cells were regularly checked for mycoplasma using MycoAlert detection kit (Lonza).
Commonly misidentified lines<br>(See ICLAC register)	No commonly misidentified cells lines were used.

Animals and other research organisms

Policy information about studies involving animals; ARRIVE guidelines recommended for reporting animal research, and Sex and Gender in Research

Laboratory animals	Laboratory mice of the appropriate genotypes were bred. Adult mice were used between 8 and 12 weeks old to derive cells and tissues. Stag2 lox (Strain #:030902, mixed C57BL/6 129 background), VavCre (Strain #035670, mixed C57BL/6 129 background) and OT-1 (Strain #003831, mixed C57BL/6 129 background) were obtained from The Jackson Laboratory
Wild animals	The study did not involve wild animals.
Reporting on sex	Sex-based analysis was used as is reported throughout the manuscript
Field-collected samples	The study did not involve samples collected from the field.
Ethics oversight	Ethical approval was granted by Home Office, UK, and a local Ethics Committee as required by the Animals (Scientific Procedures) Act.

Note that full information on the approval of the study protocol must also be provided in the manuscript.

Plants

Seed stocks	*Report on the source of all seed stocks or other plant material used. If applicable, state the seed stock centre and catalogue number. If plant specimens were collected from the field, describe the collection location, date and sampling procedures.*
Novel plant genotypes	*Describe the methods by which all novel plant genotypes were produced. This includes those generated by transgenic approaches, gene editing, chemical/radiation-based mutagenesis and hybridization. For transgenic lines, describe the transformation method, the number of independent lines analyzed and the generation upon which experiments were performed. For gene-edited lines, describe the editor used, the endogenous sequence targeted for editing, the targeting guide RNA sequence (if applicable) and how the editor was applied.*
Authentication	*Describe any authentication procedures for each seed stock used or novel genotype generated. Describe any experiments used to assess the effect of a mutation and, where applicable, how potential secondary effects (e.g. second site T-DNA insertions, mosiacism, off-target gene editing) were examined.*

ChIP-seq

Data deposition

☒ Confirm that both raw and final processed data have been deposited in a public database such as GEO.

☒ Confirm that you have deposited or provided access to graph files (e.g. BED files) for the called peaks.

Data access links<br>*May remain private before publication.*	Please use https://www.ncbi.nlm.nih.gov/geo/query/acc.cgi?acc=GSE240997 and enter the token wpcfeiygthqzfej.
Files in database submission	GSM8147518_6183_1_WT_-_SCC1_CGATGT_S28.bw<br>GSM8147519_6183_5_SAWAm_-_SCC1_ACTTGA_S32.bw
Genome browser session<br>(e.g. UCSC)	N/A

Methodology

Replicates	RAD21 ChIP was performed only once and was analysed by ChIP-Seq.
Sequencing depth	sample total_reads uniquely_mapped length type<br>GSM8147518_6183_1_WT_-_SCC1_CGATGT_S28.bw 40279851 37758949 65 single<br>GSM8147519_6183_5_SAWAm_-_SCC1_ACTTGA_S32.bw 34314496 32515989 65 single
Antibodies	RAD21: Millipore, Cat# 05-908
Peak calling parameters	RAD21 peaks were previously called (Li Y, et al. Nature. 2020). Peaks for SMC1 ChIP seq were called using MACS3 3.0.0b1 with standard settings
Data quality	SMC1 ChIPseq: 14,120/21,608 (65.35%) peaks are above 5-fold enrichment and 20,078/21,608 (92.92%) are FDR <0.05. All peaks above 5 fold enrichment are FDR < 0.05.
Software	RAD21 ChIPseq: Heat maps were generated using DeepTools on previously called RAD21 peaks (Li Y, et al. Nature. 2020).<br>SMC1 ChIP seq: peaks called with MACS3. Heatmaps were produced using the genomation toolkit

Flow Cytometry

Plots

Confirm that:

☒ The axis labels state the marker and fluorochrome used (e.g. CD4-FITC).

☒ The axis scales are clearly visible. Include numbers along axes only for bottom left plot of group (a 'group' is an analysis of identical markers).

☒ All plots are contour plots with outliers or pseudocolor plots.

☒ A numerical value for number of cells or percentage (with statistics) is provided.

Methodology

Sample preparation	For the isolation of LSKs, c-kit+ cells and CLPs, bone marrow cells were depleted of lineage markers (CD4,CD8,CD19,B220,NK1.1,Cd11b, Ter119, Gr-1, Miltenyi 130-048-102). Lineage-negative cells were stained with Sca-1-BV510, c-kit-PE-Cy7, FLT3-PE, CD127-APC and streptavidin-ef450. To isolate B cell progenitors, bone marrow cells were depleted of Ter119, Gr-1 and Cd11b. Cell were stained with B220-FITC, CD19-PE, IgM BV421 and CD43-APC antibodies. Mature monocytes and granulocytes were isolated from bone marrow cells stained with Cd11b-APC and Ly6-G-FITC antibodies. To isolate double negative (DN), double positive (DP), CD4 and CD8 single positive cells from the thymus, thymocytes were stained with CD4-BV421, CD8-APC, CD25-PE and TCRbeta-FITC. To isolate B cells and CD4 T cells from lymph nodes, lymphocytes were stained with B220-BV421 and CD4-PE.
Instrument	Cell populations were sorted using a BD Aria Fusion or AriaIII. Cell populations were analysed using a Fortessa Flow Cytometer (BD)
Software	BD FACSDiva Sotfware was used to collect data. Data was analysed using Flowjo v10.
Cell population abundance	Purity was determined by running a purity check of the sorted populations after the sort was completed.
Gating strategy	Forward and side scatter gating was used to record and collect viable singlet cells. Bone marrow progenitors were defined based on the expression of Sca-1 and c-kit (Fig. 2f i), within the lineage-negative fraction. The gating strategy for B cell

progenitors, mature monocytes and granulocytes is showed in Supplementary Fig.7. B cells and CD4 T cells were gated as shown in Fig.2c i. The gating scheme used to identify double negative (DN), double positive (DP), CD4 and CD8 single positive cells is shown in Fig. 2e i. DN cells were further gated according to their CD25 expression and CD8 single positive cells were further gated based on their TCRbeta expression.

☒ Tick this box to confirm that a figure exemplifying the gating strategy is provided in the Supplementary Information.
