# Supplementary figures and images for: Competition shapes the landscape of X-chromosome-linked genetic diversity
Source: Nat Genet. 2024 Jul 26;56(8):1678–88. doi: 10.1038/s41588-024-01840-5 (PMC11319201; doi:10.1038/s41588-024-01840-5)

Before  
processing

Wt DP

SA2-W Mut DP

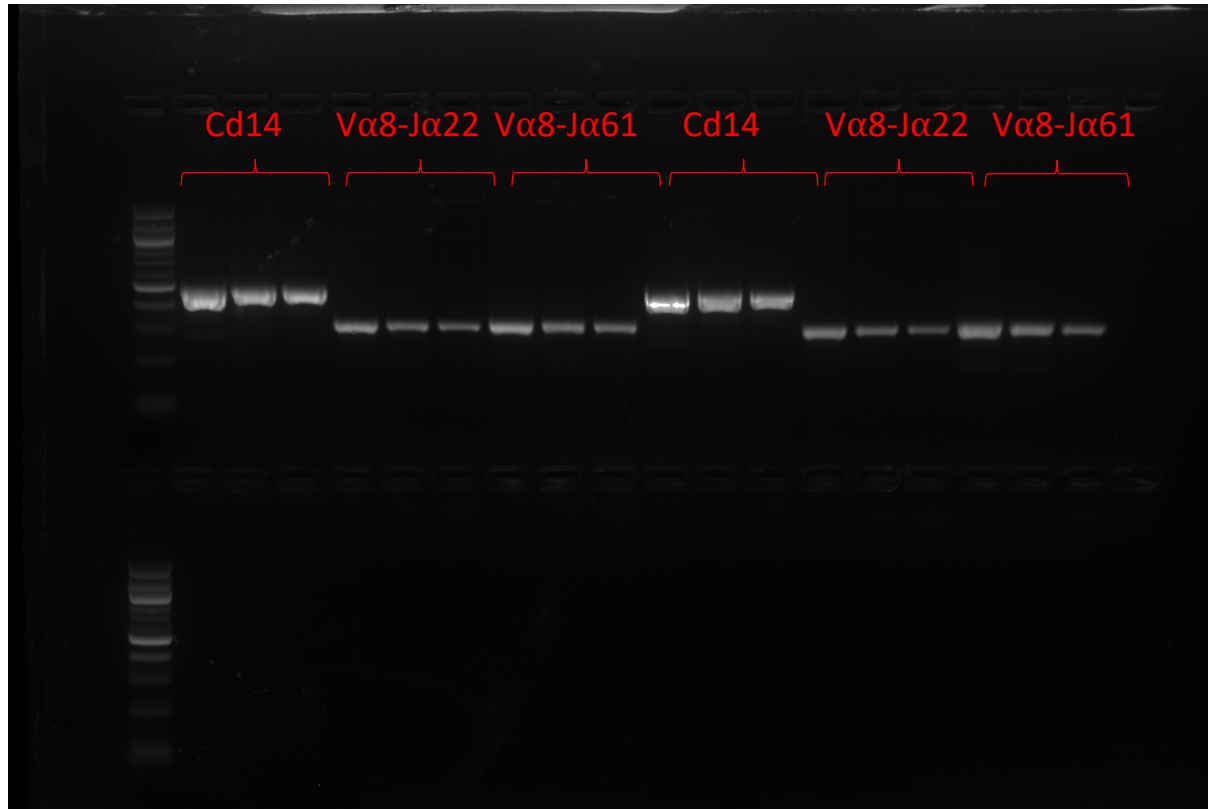

After  
processing

Wt DP

SA2-W Mut DP

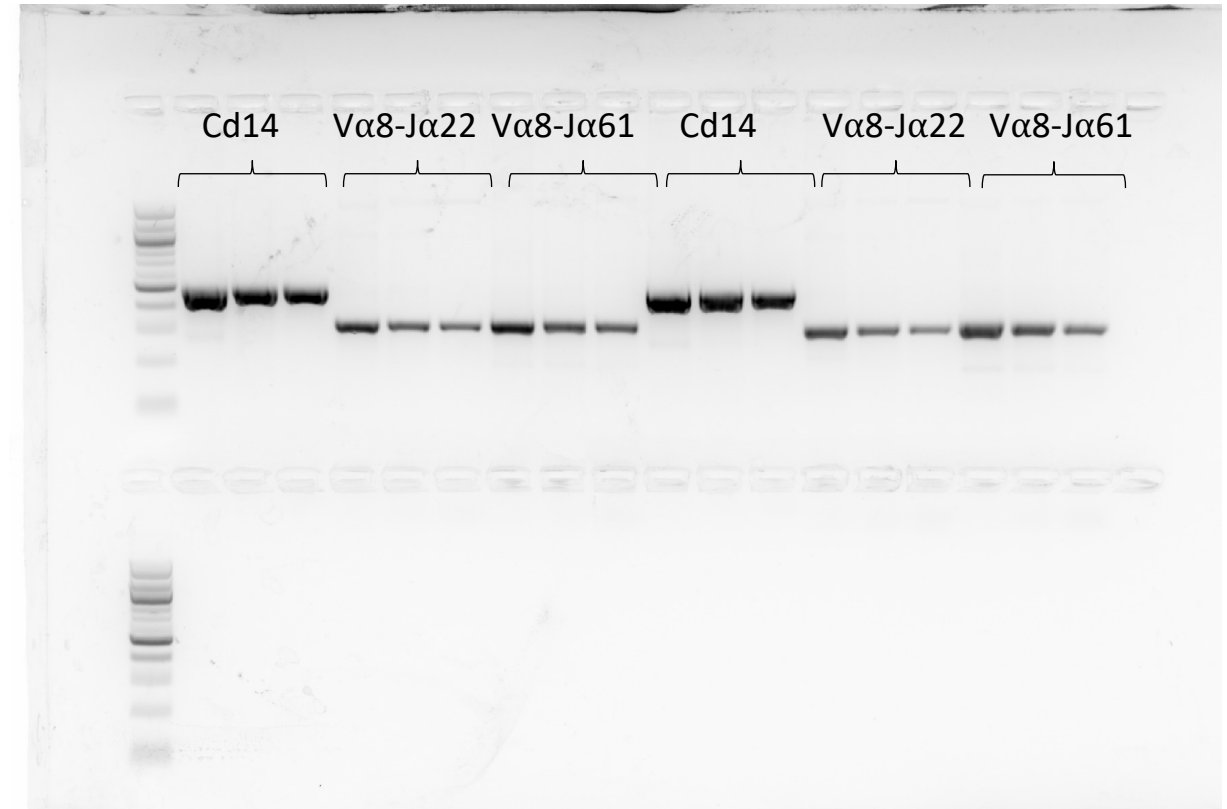

Supplement: Supplementary file 15 — Unprocessed and processed gel. [file 41588_2024_1840_MOESM15_ESM.pdf]
